# The Insecticide Imidacloprid Causes Mortality of the Freshwater Amphipod *Gammarus pulex* by Interfering with Feeding Behavior

**DOI:** 10.1371/journal.pone.0062472

**Published:** 2013-05-15

**Authors:** Anna-Maija Nyman, Anita Hintermeister, Kristin Schirmer, Roman Ashauer

**Affiliations:** 1 Department of Environmental Toxicology, Eawag - Swiss Federal Institute of Aquatic Science and Technology, Dübendorf, Switzerland; 2 Department of Environmental Systems Science, ETH Zürich, Zürich, Switzerland; 3 School of Architecture, Civil and Environmental Engineering, EPF Lausanne, Lausanne, Switzerland; 4 Environment Department, University of York, Heslington, York, United Kingdom; Indian Institute of Toxicology Research, India

## Abstract

If an organism does not feed, it dies of starvation. Even though some insecticides which are used to control pests in agriculture can interfere with feeding behavior of insects and other invertebrates, the link from chemical exposure via affected feeding activity to impaired life history traits, such as survival, has not received much attention in ecotoxicology. One of these insecticides is the neonicotinoid imidacloprid, a neurotoxic substance acting specifically on the insect nervous system. We show that imidacloprid has the potential to indirectly cause lethality in aquatic invertebrate populations at low, sublethal concentrations by impairing movements and thus feeding. We investigated feeding activity, lipid content, immobility, and survival of the aquatic arthropod *Gammarus pulex* under exposure to imidacloprid. We performed experiments with 14 and 21 days duration, both including two treatments with two high, one day pulses of imidacloprid and one treatment with a low, constant concentration. Feeding of *G. pulex* as well as lipid content were significantly reduced under exposure to the low, constant imidacloprid concentration (15 µg/L). Organisms were not able to move and feed – and this caused high mortality after 14 days of constant exposure. In contrast, feeding and lipid content were not affected by repeated imidacloprid pulses. In these treatments, animals were mostly immobilized during the chemical pulses but did recover relatively fast after transfer to clean water. We also performed a starvation experiment without exposure to imidacloprid which showed that starvation alone does not explain the mortality in the constant imidacloprid exposure. Using a multiple stressor toxicokinetic-toxicodynamic modeling approach, we showed that both starvation and other toxic effects of imidacloprid play a role for determining mortality in constant exposure to the insecticide.

## Introduction

To protect crops and seeds from pests, about 3 billion tons of pesticides are applied annually to fields worldwide [1]. A fraction of this reaches other environmental compartments such as surface waters via runoff, spray drift and leaching. One of the world’s best-selling insecticide is imidacloprid, 1-(6-chloro-3-pyridylmethyl)-*N*-nitroimidazolidin-2-ylideneamine, which belongs to the chemical group of neonicotinoid insecticides [2]. Neonicotinoids have selective toxicity for insects and act by binding to the nicotinic acetylcholine (ACh) receptors in the receiving nerve cells of the central nervous system [3], [4]. Mammals have lower numbers of nicotinic receptors with high affinity to neonicotinoids, which is why the toxicity of these insecticides is low in mammals [5]. Imidacloprid has a relatively high water solubility (610 mg/L in 20°C H_2_O; log *K*_ow_ = 0.57) and therefore, a great potential to reach water bodies. Accordingly, several studies have reported the occurrence of imidacloprid in surface waters [6], [7] where it may affect non-target organisms such as *Gammarus pulex* (Crustacea, Amphipoda, Gammaridae). The concentrations of imidacloprid in surface waters in Sweden reported by Kreuger and coworkers (max. 15 µg/L) [7] are below lethal acute toxicity levels in *G. pulex* (50% of the test individuals die after constant exposure to 270 µg/L for 4 days [8]). However, the lower concentrations found in water bodies might cause sublethal effects.

In aquatic environments pesticide contamination generally occurs in pulses due to fluctuation in rainfall, seasonal application of pesticides, and accidents [9], [10]. Because neonicotinoids lack ester bonds and thus cannot be hydrolyzed by ACh esterase, also temporary exposure to these insecticides can generate sustained activation in receptors and cause long lasting effects. However, it has been shown that imidacloprid can be dissociated (dissociation constant 0.419 min^−1^) and removed from ACh receptors by ACh and other ligands [11]. Therefore, it is possible that organisms recover between imidacloprid pulses. On the other hand the elimination of imidacloprid in *G. pulex* is very slow [12] and the substance is not biotransformed [13]. Thus, one could also expect cumulative effects from subsequent exposure events. Imidacloprid has not shown cumulative effects on *Gammarus roeseli* survival after repeated pulses of the insecticide [14]. However, a cumulative sublethal effect (increased drifting) has been reported [15].

By binding to the ACh receptors and interfering with nerve impulses, imidacloprid causes twitching, cramps and muscle weakness. Therefore, it impairs invertebrate movements and can lead to starvation and death via dysfunctional feeding behavior. It has been shown previously that imidacloprid inhibits feeding of many non-target aquatic species [16]–[18]. However, the connection between impaired feeding and mortality via starvation has not been further investigated, in spite of the importance of conserving populations of aquatic shredding invertebrates like *Gammarus pulex*.

We studied the effects of imidacloprid on feeding rate, lipid content, immobility and survival of *G. pulex* in 14-day and 21-day long experiments. As exposures in aquatic environments generally occurs in pulses, we exposed the animals to two high imidacloprid concentrations. To further study the impact of imidacloprid under low constant exposure, we also exposed *G. pulex* to the time weighted average concentration, which was 15 µg/L. Using the time weighted average concentration allowed us to compare effects of different exposure patterns while still employing the same overall dose (time × concentration). We chose the low concentrations for the constant treatments [8], because we hypothesized that starvation causes death in constant treatments due to impaired movements. In the pulsed treatments, however, feeding activity might recover between chemical pulses and thus starvation would not play a big role in determining mortality. In contrast, we can observe other toxic effects of imidacloprid, e.g. direct mortality, in response to high imidacloprid peaks. Thus, there might be different mechanisms behind mortality in pulsed and constant treatments. To investigate this, we used toxicokinetic-toxicodynamic (TKTD) modeling to analyze the survival data and tested whether fitted model parameters indicate different effect mechanisms in the pulsed and constant exposures. We performed also a starvation experiment, without adding imidacloprid, to further investigate the effect of starvation on survival and to test if using a calibrated starvation model would predict survival in our constant imidacloprid treatments. The chemical effect and the starvation models were also combined to develop a multiple stressor model which again was tested by simulating survival in constant exposure treatments.

## Materials and Methods

### Test Animals and Chemicals

*Gammarus pulex* is an important invertebrate species in lentic waters for e.g. decomposition of organic material and nutrient cycling [19]. The *G. pulex* test individuals in our study were collected from a small headwater stream in the Itziker Ried, Switzerland (E 702150, N 2360850). No permission to collecting was required as *G. pulex* is not an endangered species and the site is located on public land. The test animals were maintained for 5–7 days prior to the experiments in a large aquarium in a temperature controlled room (13°C, 12∶12 light:dark photoperiod) and were fed with horse chest-nut (*Aesculus hippocastanum*) leaves which were inoculated with the fungi *Cladosporium herbarum* for at least 10 days [20]. The water in the aquarium was preaerated artificial pond water (APW, Table S1 in File S1).

^14^C-labelled imidacloprid (radiochemical purity 96.97%) was purchased from the Institute of Isotopes Co., Ltd. Budapest, Hungary and unlabeled material (chemical purity 99.9%) from Sigma-Aldrich. A mixture of both was dissolved in acetone and used for dosing.

### Imidacloprid Experiments

A 14-d and a 21-d toxicity experiment including three treatments plus controls in each were conducted. Two of the treatments (A, B) included two 1-day imidacloprid pulses with differing recovery time between pulses in uncontaminated APW. In another treatment (C), the concentration was maintained constant (15 µg/L, 0.06 µmol/L in both experiments) but the overall dose was the same over time as in the pulsed treatments (i.e. time-weighted average concentration). All treatments included 7 replicate beakers, one plain and one solvent control beaker. Each 600 mL Pyrex beaker contained 500 ml of APW and 5 leaf discs (diameter of 20 mm, *Aesculus hippocastanum* leaves inoculated with *Cladosporium herbarum*). Ten *G. pulex* were placed in each beaker a day prior to the experiments. The beakers were covered with parafilm and kept in a climate chamber (13°C, 12∶12 light:dark photoperiod). The beakers were spiked individually and after spiking, test solutions were stirred with a glass rod and 1 mL samples were taken from the solution to quantify the initial chemical concentration in medium. Ten mL of Ecoscint A scintillation cocktail (Chemie Brunschwig, Switzerland) were added to the samples and activities were counted using a liquid scintillation counter (LSC, Tri-Carb 2200CA, Packard, USA). Samples to determine imidacloprid concentrations in water were taken throughout the experiments (see time points and concentrations in Tables S2 and 3 in File S1). Only the total radioactivity in the aqueous samples was measured and therefore imidacloprid could not be differentiated from its possible breakdown products. For example, organisms might be exposed to the breakdown products of imidacloprid rather than the parent compound due to fast photolysis of imidacloprid in aqueous medium with a half-life of 1.2 h at 290 nm irradiation [21]. However, it is also shown that wavelength has a great impact on the photolysis and already in 365 nm, the half-life is extended to 18 h [22]. The wavelengths in our experiments resemble those of day light ranging from 380–730 nm (relative intensity being the highest in 580 nm). Thus under the conditions of our experiments, imidacloprid most likely is less susceptible to photolysis and in an earlier study no breakdown products of imidacloprid were observed in *G.pulex* samples [13]. The test solution was changed at least every 5 days. Always during water change and every time when eaten leaf discs were observed, they were replaced by new ones. Water pH, conductivity and oxygen concentration were measured in exposed and non-exposed conditions during experiments (see in more detail in Tables S4 and S5 in File S1).

In the 14-day experiment mortality, immobility and consumption of leaf discs were observed. In addition, internal concentrations of imidacloprid in *G. pulex* were measured. The pulsed treatments (A, B) had 4 (A) and 8 (B) days between 1-day pulses (concentration 90 µg/L = 0.35 µmol/L) and individuals in treatment C were exposed constantly to a concentration of 15 µg/L (0.06 µmol/L). Immobility was defined as incapability of moving after ten gentle prods with a glass rod. Immobile individuals were taken out of the beakers and frozen until analysis of internal concentrations. In addition, mobile individuals were sampled for analysis of internal concentrations at the end of the experiment (A and B: *n* = 21 per treatment, C: *n* = 10) and during the experiment from additional beakers of the treatments A and B. These supplemental beakers were not used for observation of survival, immobility and consumption of leaf discs. See detailed sampling times and internal concentrations in Tables S6, S7, S8 in File S1.

Sample processing and quantification of radioactivity in *G. pulex* were measured similarly to Ashauer and co-workers [12]. In short, individuals were plotted dry with tissue paper, weighed in pre-weighed glass vials and frozen in −20°C. For analysis, 3 mL of the tissue solubilizer Soluene-350 (Perkin Elmer, USA) was added to vials. Vials were placed in a water bath (60°C, 24 h) and after cooling down, 15 mL of scintillation liquid Hionic Fluor (Perkin Elmer, USA) was added. Radioactivity was counted using a liquid scintillation counter; color quenching and efficiency were corrected using external standards and background activity (i.e. activity in control samples was subtracted from counts of the samples).

The second, 21-day long experiment included also observations of survival, immobility and food consumption. In addition, lipid content was measured from immobile individuals, which were sampled and frozen any time they were observed as well as from mobile individuals sampled at the end of the experiment. The pulsed treatments (A, B) had 4 (A) and 11 (B) days between 1-day pulses (concentration 140 µg/L = 0.59 µmol/L) and individuals in treatment C were exposed constantly to a concentration of 0.06 µmol/L.

### Feeding Rate

In the 14-day experiment, food consumption was measured as the number of leaf discs consumed by *G. pulex* individuals in each beaker. Every time when a leaf disc was fully eaten, it was replaced by a new leaf disc and the exchange was noted. This way of measuring was not based on the mass of leaf discs but only on the number of same sized discs. During the 21-day experiment food consumption was measured as the mass of leaf material. Wet weight of the leaf discs of each beaker was measured before providing them to the test organisms and when removing the rest of them from the beakers. Food consumption is given as cumulative amount consumed over time (either amount of leaf discs (14-day experiment) or mg (21-day experiment)). The food consumption was divided by the number of mobile organisms in the respective beaker (amount consumed/*G. pulex*). Statistical testing to compare feeding among treatments was performed for cumulative food consumption at the end of the experiments using the non-parametric Kruskal-Wallis test and further pairwise testing with the Wilcoxon rank sum test. The assumption of normality could not be tested due to the too small sample size (i.e. number of replicate beakers, 7 per treatment) and thus a normal distribution of the data could not been assumed and one-way analysis of variance could not been used. Analyses were performed using the software R (www.r-project.org).

### Lipid Content

**Figure 1:**
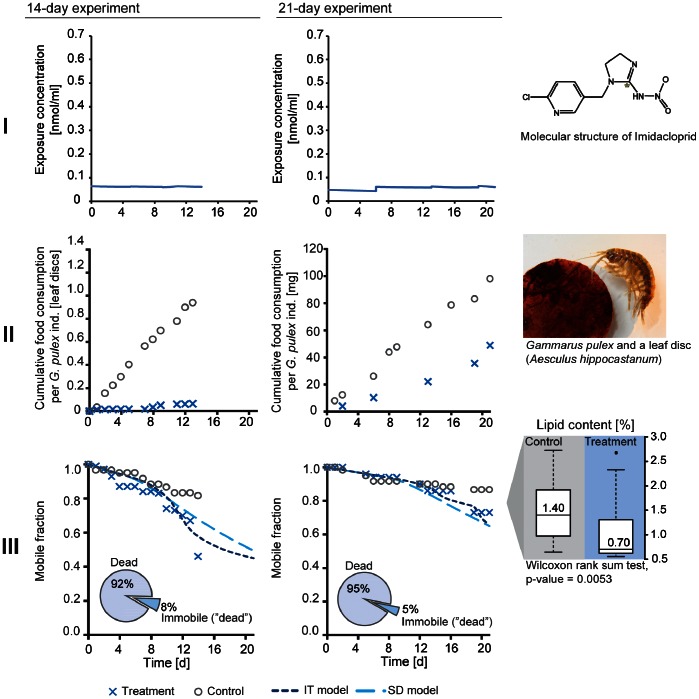
Feeding, lipid content, and survival of *Gammarus pulex* under constant exposure to imidacloprid. Imidacloprid concentrations in medium (I), cumulative food consumption (II), mobile fraction of individuals, and lipid content (% of total wet weight) of *Gammarus pulex* (III) in the constant treatments (C) and controls of 14-day and 21-day experiments. Pie charts show the percentage of dead and immobile individuals amongst those removed from the beakers (non-mobile individuals = immobile+dead).

The lipid content was analysed from immobile *G. pulex* sampled during the 21-day experiment (treatment A: 33 samples, treatment B: 28 samples, treatment C: 1 sample) and mobile individuals sampled at the end of the experiment (*n* = 21 for each treatment, total of 30 for controls: 5 from plain and 5 from solvent control beaker of each treatment). A gravimetric method was used to determine the lipid content according to Kretschmann and coworkers [23]. In short: Extraction was done using H_2_O, i-PrOH, and cyclohexane (11∶8:10) in 2 mL Eppendorf tubes. 300 mg of zirconia/silica beads (Ø 0.5 mm, BioSpec Products, Bartlesville, OK, USA) was added to iPrOH/cyclohexane solution together with the sample and FastPrep® FP120 Bio 101 (Savant Instruments, Inc., NY, USA) was used to break down the tissues of *G. pulex*. Nanopure water was added to samples. A water content of 77% of *G. pulex* wet weight was assumed to achieve a 11∶8:10 ratio of H_2_O, i-PrOH, and cyclohexane. Then, samples were vortexed, centrifuged (20 min, 450 g, 20°C) (Centrifuge 5417R, Vaudaux-Eppendorf AG, Schönenbuch/Basel, Switzerland) and the organic phases were separated. Volumes of 435 µL of cyclohexane and 65 µL of iPrOH were added once more, and after vortexing, centrifugation and separation of the organic phase, the solvents of the combined organic phase aliquots were evaporated under nitrogen flow and extracts were dried at 60°C for 14 hours. The remaining phases, i.e. the lipids, were weighted. The weight of lipids was divided by total wet weight to obtain the lipid content as a percentage. Because the lipid content in treatment C did not follow a normal distribution (see Figure 1 and Figure S1, raw data are provided in Table S11 in File S1), the differences among treatments at the end of experiment were compared by the Kruskal-Wallis rank sum test and further pairwise testing (control-treatment) using the Wilcoxon rank sum test. The software R was used for the analysis.

### Chemical Stress Modeling

The survival/mobility data of the imidacloprid experiments was analyzed using the toxicokinetic-toxicodynamic (TKTD) model GUTS (General Unified Threshold model for Survival) published by Jager and co-workers [24]. The model was implemented in the software ModelMaker 4 (Cherwell Scientific Ltd., Oxford, UK). The fraction of mobile animals over time was used to calibrate the model. Note that immobile animals were removed from the experiment, allowing us to apply the same assumptions about error structure to our immobility data as to survival data and to use GUTS for modeling the mobility/survival data. As we suspect that constant low concentration of imidacloprid (treatments C) might have a different mechanism for survival/mobility than the pulsed treatments (A and B), the TKTD model was calibrated separately using the constant treatments C of both experiments together and the pulsed treatments (A, B) of both experiments together. In addition, the model was fitted to all data in order to compare the parameter estimates with those of the separately fitted data sets. The parameter estimates from the fit to all data were used as initial values for fitting the model separately to pulsed and constant treatments.

Imidacloprid is not biotransformed in *G. pulex* [13]. Therefore, a one-compartment toxicokinetic model (Eqn 1) was used to simulate the internal concentration of imidacloprid.
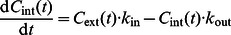
(1)where *C*_int_ (t) is the internal imidacloprid concentration in organisms [µmol/kg], *C*_ext_ (t) is the concentration in water [µmol/L], *k*_in_ is the uptake rate constant [L⋅kg^−1^⋅d^−1^], *k*_out_ is the elimination rate constant [1/d] and *t* is time [d]. Uptake and elimination rate constants were estimated by Ashauer and co-workers (*k*_in_: 1.96 [L⋅kg^−1^⋅d^−1^]; *k*_out_: 0.267 [1/d]) [12]. We validated this TK model by comparing its predicted internal concentrations with measured internal concentrations in our first experiment.

Survival modeling was based on the GUTS model and the modeling is similar to our previous study with *G. pulex* and propiconazole [25]. However, here least squares optimization together with the Marquardt algorithm was used to fit models to the data. Confidence intervals (95%) were calculated from standard errors as described in Motulsky & Christopoulos 2003 [26]. Fitting was also performed by maximizing the log-likelihood function, which maximizes the likelihood of yielding the parameter set which best describes the number of death events between time intervals (see Table S14 in File S1). In this study, the cumulative fraction of survivors over time was better described by the parameters found via least squares optimization.

Two models, either assuming stochastic death (SD) or individual tolerance distribution (IT) were used separately to test which hypothesis of death applies for imidacloprid. SD models have one value for the threshold of survival and after exceeding it, an organism has an increased probability to die. In contrast, according to IT models the threshold is distributed within the population and death is instantaneous after exceeding the individual threshold. To date, it is not known which of the two hypotheses describes our data better (see also discussion in [24] and [25]). Therefore, both models, SD and IT, were calibrated and the goodness of fit values were compared.

Equation 1 was used to simulate the internal concentrations (*C*_int_) in the survival model for both, GUTS-SD and GUTS-IT. The implementation of the stochastic death model (GUTS-SD) is given in Eqns 2 to 5. Eqns (2) and (3) were used to calculate the cumulative hazard at time *t* (*H*(*t*)).
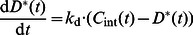
(2)

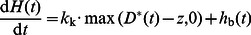
(3)where *D* ^*^ (t) is the scaled damage [µmol/kg], *k*_d_ is the damage recovery [1/d], *k*_k_ is the killing rate [kg ⋅ µmol^−1^ ⋅ d^−1^], *H* (t) is the cumulative hazard of an individual [−], *z* is the threshold for effects [µmol/kg], *h*_b_ is the background hazard rate [1/d] (Eqn 4) and the ‘max’ function selects the maximum of either 0 or (D*(t) − *z*). The background hazard rate *h*_b_ was obtained by fitting Eqn 4 to survival data of plain and solvent controls combined.
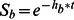
(4)where Sb is the background survival probability [−] describing survival in unexposed conditions.

Once the cumulative hazard *H(t)* is obtained, the survival probability, *S* (t) [−], was calculated using Eqn 5.
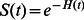
(5)


The model that assumes the threshold for death to be drawn from an individual tolerance distribution (GUTS-IT model) is presented in Eqns 6 and 7. The IT model uses the same dose metric, scaled damage *D* ^*^, as the SD model (Eqn 2). Cumulative threshold distributions are based on a log-logistic cumulative distribution function (Eqn 6). The resulting survival probability is given by Eqn 7.
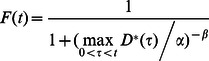
(6)

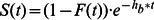
(7)where *F*(t) is the log-logistic cumulative distribution function for the threshold [−], *α* is the median of the distribution [µmol/kg], *β* determines the width of the distribution [−] and the ‘max’ function selects the largest value of the dose metric D* that occurred until time *t*.

To compare the goodness of fit among models and calibration data sets, the mean percentage error (MPE) was calculated (Eqn 8) [25]. The MPE was calculated for each treatment separately and therefore for each treatment the goodness of fit could be compared between a) stochastic death and individual tolerance models and b) models calibrated with different data sets (i.e. pulsed or constant exposures).
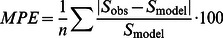
(8)where MPE is the mean percentage error [%] of the fraction of survivors, *S*_obs_ is the observed fraction of survivors [−], *S*_model_ is the model prediction of the fraction of survivors [−] and *n* is the number of data points used in the calculation.

### Starvation Experiment and Modeling

As we hypothesise that organisms in the constant imidacloprid exposure die due to impaired movements leading to starvation, an experiment studying the effect of starvation, without exposure to imidacloprid, on survival of *G. pulex* was conducted. There were 30 replicates for both, the control and the starvation treatment. In the control group, one leaf disc was provided for food and more was given when the disc was eaten. In the starvation treatment, no food was given. *G. pulex* were placed individually in 100 mL beakers in order to prevent cannibalistic behavior of the test animals, which is more likely without leaf discs. Mortality was monitored for 34 days, i.e. long enough to observe mortality of at least half of the animals. Experimental water was APW (Table S1 in File S1) which was renewed weekly.

The mortality observed in this experiment was compared with mortality under the low, constant exposure to imidacloprid. For easier comparison, modified versions of the TKTD models described above (SD and IT) were calibrated using the starvation data and the survival in the constant imidacloprid treatments was predicted using this new starvation model. Instead of using chemical internal concentration causing the scaled damage *D* ^*^ (t), a new concept, lack of food (*LF*), leading to the damage (Eqn 9) was introduced. In other words, the dose metric is *LF*, defined as the relative lack of food compared to control conditions (*LF = *1 - (available food/food available in control)). Thus the survival model for starvation consists of equations identical to Eqns 3–7, Eqn 2 being replaced by Eqn 9 (*LF* replaces *C*_int_(*t*)), which also leads to different dimensions of the model parameters. The *LF* was set to 1 when calibrating the model with starvation data as well as simulating the survival in the constant imidacloprid treatment in the 14-day experiment where hardly any food consumption was observed. When simulating the survival in 21-day imidacloprid experiment, the *LF* was set to 0.5 as food consumption in this experiment appeared to be only partially inhibited (the feeding activity was approximately half of control levels, see Figure 1).
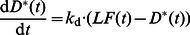
(9)where *D* (t) describes the damage caused by lack of food [−], *k*_d_ is damage recovery rate [1/d] and *LF* (t) is lack of food [−]. Units of the following parameters in Eqns 3–7 were therefore different from described above: *k*_k_ [1/d], *z* [−] and *α* [−]. The background hazard rate was calibrated for each experiment separately.

### Multiple Stressor Modeling

The starvation model was combined with the chemical stress model and the survival in constant imidacloprid exposure was simulated in order to test whether survival is determined by both, the effect of starvation and other toxic effects of imidacloprid. The chemical stress models were the GUTS models described above (Eqns 1–7) calibrated with the data from the pulsed toxicity treatments alone. In this model the effect of starvation is excluded from other chemical effects because we can assume that in the pulsed toxicity treatments starvation does not play a role due to possible recovery of the movements and feeding between chemical pulses. To implement the multiple stressor model, equations for both, chemical stress (SD: Eqns 1–4; IT: Eqns 1–2, 6) and starvation (SD: Eqns 9, 3–4; IT Eqns 9, 6) were followed. Then the cumulative hazards *H* (t) of both chemical and starvation stress were added (SD model, Eqn 10) or both cumulative distribution functions for the threshold *F* (t) were subtracted (IT model, Eqn 11) to predict survival in the constant imidacloprid treatments where both processes likely play role.
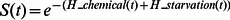
(10)

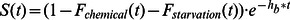
(11)


## Results and Discussion

### Feeding Rate

**Figure 2:**
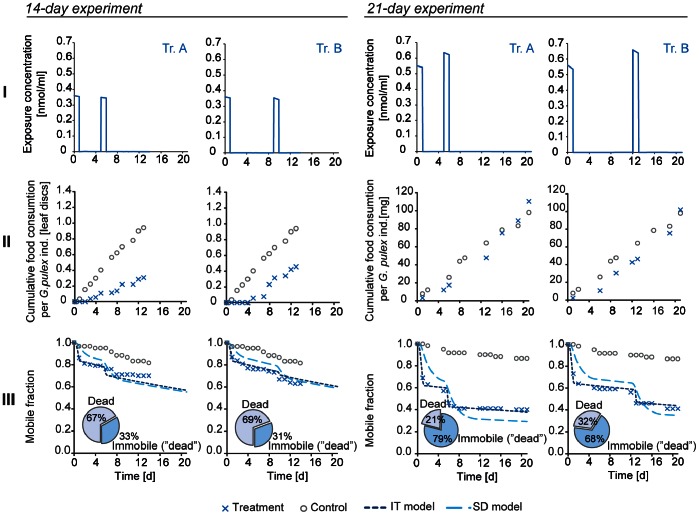
Feeding and survival of *Gammarus pulex* under pulsed exposure to imidacloprid. Imidacloprid concentrations in medium (I), cumulative food consumption (II), and mobile fraction of *Gammarus pulex* (III) in the pulsed treatments (A, B) and controls of 14-day and 21-day experiments. Pie charts show the percentage of dead and immobile individuals amongst those removed from beakers (non-mobile individuals = immobile+dead).

**Figure 3:**
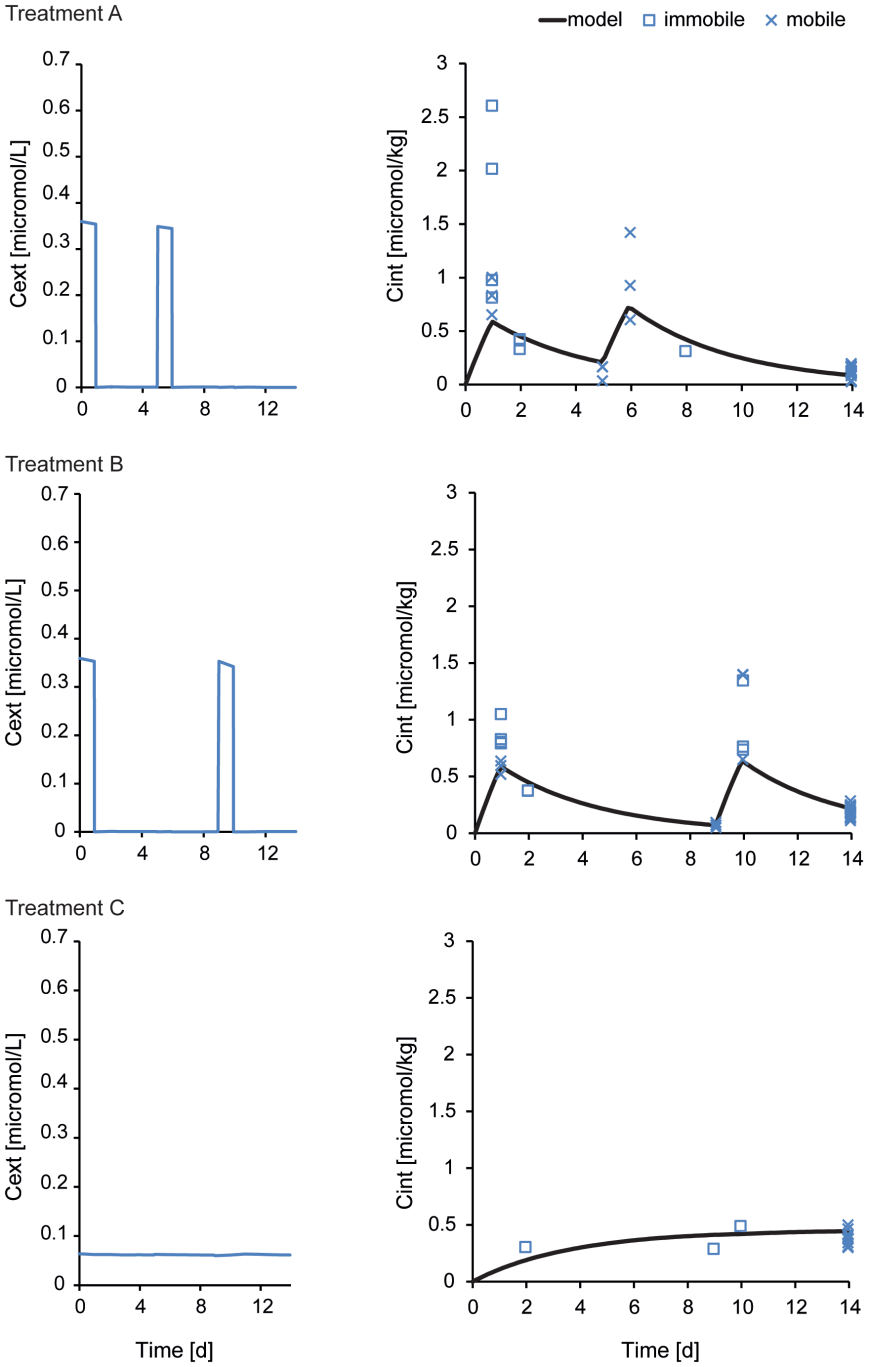
Toxicokinetic model validation. Internal concentrations were measured from immobile individuals in 14-day experiment (open squares) and from mobile individuals in additional beakers which were not used for observing mortality (crosses). These values are plotted with predictions of internal concentration (black line) by a previously published and calibrated toxicokinetic model [1].

Inhibition of feeding by imidacloprid has been observed in many invertebrate species [16]–[18], [27]–[29]. We observed that feeding of *Gammarus pulex* was heavily inhibited by imidacloprid in the constant treatment of the 14-day experiment (Figure 1, Table 1, Table S9 in File S1) while in the pulsed treatments the effect was not as strong (Figure 2, Table 1, Table S9 in File S1). However, in this experiment, the method of measuring feeding activity was only semi-quantitative because it was based on the number of same-sized, however undefined by their mass, leaf discs consumed by *G. pulex* individuals. Thus, we focused on the 21-day experiment to draw conclusions with regards to the effects of imidacloprid on feeding activity. In the 21-day experiment, feeding activity was measured as the mass of leaf material eaten [mg]. There the effect on feeding in the constant treatment was again evident, however, not as strong as in the 14-day experiment (Figure 1, Table 1, Table S10 in File S1). In the pulsed treatments, no effects were observed on feeding activity - the organisms started to feed roughly 2 days after they were transferred to uncontaminated media (Figure 2, Table 1, Table S10 in File S1). This finding is in agreement with the fact that imidacloprid binding to ACh receptors in insect membranes is reversible, i.e. it can be dissociated as well as removed by ACh and other ligands [11], [30], [31]. However, in spite of the fairly fast dissociation of imidacloprid from the ACh receptors (0.419 min^−1^ [11]), the elimination of imidacloprid has been shown to be slow in *G. pulex* [12] and can be associated with the receptors again. The internal concentrations in our experiments decrease relatively fast (Figure 3) and 2 days after a pulse around 60% of imidacloprid is left inside the organisms. Once feeding activity is recovered in 2 days, it seems that the internal concentrations during these days fall below the threshold of preventing animals from feeding.

**Table 1:** Feeding activity of *Gammarus pulex* under constant (treatment C) or pulsed (treatments A and B) exposure to imidacloprid.

				Statistics (*p*-value)	
Experiment	Treatment	Median cumulative feeding	Units	Wilcoxon test^4^	Kruskal-Wallis test^5^
14-day	Control	0.929	leaf discs/*G.pulex*		0.0010
14-day	A (pulsed)^1^	0.286	leaf discs/*G.pulex*	0.010	
14-day	B (pulsed)^2^	0.500	leaf discs/*G.pulex*	0.031	
14-day	C (constant)	0.000	leaf discs/*G.pulex*	0.003	
					
21-day	Control	103.9	mg/*G.pulex*		0.0023
21-day	A (pulsed)^1^	114.2	mg/*G.pulex*	0.234	
21-day	B (pulsed)^3^	104.4	mg/*G.pulex*	0.731	
21-day	C (constant)	44.75	mg/*G.pulex*	0.002	

1 Pulsed treatment with a short interval in uncontaminated water between imidacloprid pulses (4 days).

2 Pulsed treatment with a long interval in uncontaminated water between imidacloprid pulses (8 days).

3 Pulsed treatment with a long interval in uncontaminated water between imidacloprid pulses (11 days).

4 Between control and treatment.

5 Among all treatments within one experiment.

The ability to recover from imidacloprid pulses has been observed also by other authors [17], [18], [32]. Alexander and co-workers [17] observed that both mayfly (*Epeorus longimanus*) larvae and the oligochaete worm (*Lumbriculus variegatus*) could recover from 1-day exposure to imidacloprid in 4 days and the recovery potential was concentration dependent [17]. Concentration dependency was not observed in this study – in fact in the 21-day experiment where we had higher imidacloprid pulses, the feeding rate was not different from the controls while in the 14-day experiment we observed an effect. However, this observation could be caused by differences in the measurement method (number of leaf discs versus mg) and variation in organism fitness between the experiments, which can be seen for example in the control mortality (Figure 1).

### Lipid Content

We observed reduced lipid content in *G. pulex* after exposing them constantly to imidacloprid for 21 days (21-day experiment, Figure 1). In the pulsed treatments, lipid content was not different from that of the controls (medians of A: 1.36%, B: 1.45%, Figure S1). When comparing among all treatments using the Kruskal-Wallis rank sum test, the *p*-value of 0.017 indicated significant differences and the pairwise comparisons showed that the constant treatment was responsible for the difference (Wilcoxon rank sum test between control and C: *p* = 0.0053). The differences in lipid content between pulsed treatments and controls were not significant (A: *p* = 0.4646; B: *p* = 0.6834). The decreased lipid content was observed in the same treatment where the feeding was inhibited (C). Therefore, we can conclude that lipid content is affected by a decreased feeding rate and might imply starvation of *G. pulex* in the presence of imidacloprid.

### Mortality and Immobility due to Imidacloprid

We observed a sudden drop in survival in the end of the constant treatments (C), especially in the 14-day experiment (Figure 1, part III, Tables S12 and S13 in File S1). The percentage of dead out of all immobile individuals (dead+only immobile) was high in the constant treatments. In fact, we did not observe many individuals classified as immobile in the constant treatment (i.e. 14-day experiment: 3 out of 70, 21-day experiment: 1 out of 70). However, almost all the “mobile” animals were close to the limit of immobility, they were passive and could not move in a normal way. Similar behavior has been observed before in *Gammarus roeseli* exposed to 12 µg/L imidacloprid [15] which is close to the concentrations in our constant treatments. This effect of imidacloprid decreased the feeding rate and might have caused starvation in our experiments. In our treatments with high imidacloprid pulses, the organisms were mostly immobile (Figures 1 and 2), thus even in the highest concentration that we used (0.59 µmol/L), the acute lethal toxicity was not reached within one day. There were no differences in the mobile fraction at the end of both experiments between treatments with short (A) and long (B) recovery time between pulses, also not in feeding activity and lipid content in the 21-day experiment (Figure 2, Figure S1). This indicates that organisms recovered fast from imidacloprid exposure between the pulses, even in the treatments A which had short intervals between pulses. Calculated 95% organism recovery times were 12.7 and 12.3 days according to IT and SD models. Organism recovery times were calculated as the time when the modeled internal damage has dropped to 5% from the maximum in a pre-defined exposure scenario [25], [33], [34]. Note that organism recovery refers to the recovery of the underlying damage that causes effects on survival until it reaches levels far below those causing mortality. Thus, the observed fast recovery with regards to mortality after the pulses does not necessarily conflict with organism recovery times of 12 to 13 days.

We observed more mortality in the constant treatment of the 14-day experiment than in the constant treatment of the 21-day experiment, even though the exposure concentration was the same and the animals were exposed longer in the 21-day experiment. This variation in our results was also seen in the background mortality: during the 14-day experiment, mortality in the controls was much higher (Figure 1). This observation can be explained by organism fitness, which varies when we collect animals from the field for each experiment. For instance, season has been shown to influence the condition of gammarids [35]–[37]. One important seasonal factor is food availability. After leaf fall during autumn, organisms have shown to have better lipid reserves during winter (maximum for males in November and for females in January) while during summer lipid reserves are the lowest due to scarcity of food [35]. This can make summer populations more sensitive to toxicants [37], especially to imidacloprid which interferes with feeding behavior.

We conducted the experiments in November (14-day experiment) and February (21-day experiment) – both are months of the season where we expect high lipid contents according to Stroda and Cossu-Leguille [35]. We can hypothesize that in November, when we conducted the 14-day experiment resulting in high mortality in the constant treatment, the organisms had not had enough time to build up and store lipids after the leaf fall. Thus, in our 21-day experiment, organisms possibly had better lipid reserves, which might be why less individuals died than in the 14-day experiment. An alternative explanation could be pre-exposure of the animals to pollutants in the field before collection, which is more likely in November and the weeks before. Although the collection site is in a headwater stream with low probability for pollution, it cannot be ruled out that some exposure occurred, for example to pesticides applied in autumn and transported via runoff in autumn rains. Independent of the possible causes, the variance in background mortality among experiments was corrected in our survival models by using experiment specific background hazard rates (Eqn 4, 14-day experiment: 0.0144 [1/d]; 21-day experiment: 0.0079 [1/d]).

From our survival models, stochastic death (SD) and individual tolerance distribution (IT), the IT model fitted better to the data (Table 2). The mean percentage error (MPE) in the treatments was always below 5.4% in the IT models (mean 4.3±1% when all data was used for calibration) while in the SD models the maximum error was as high as 22.7%, with a mean of 10.3±6.8% when all data was used for calibration. This would imply that the data provided here supports the individual tolerance distribution hypothesis, which assumes that organisms have individual effect doses which are distributed within a population. A possible interpretation is the variability of lipids and other energy reserves within the population. Similar and opposing findings, i.e. studies where the SD hypothesis described the data better, have been published earlier [34], [38]–[40]. The applicability of either extreme hypothesis seems to be chemical and species specific. Because our data supports the IT theory, we use the IT model results to compare survival among pulsed and constant treatments. The toxicokinetic sub-model calibrated by Ashauer and coworkers [12] predicted well the internal concentrations, which were measured in the 14-day experiment (i.e. model validation, see Figure 3).

**Table 2:** Mean percentage error (%) of individual tolerance (IT) and stochastic death (SD) model when pulsed (PT), constant (CT), or all data was used for calibration of the survival model for *Gammarus pulex* exposed to imidacloprid.

		Pulsed treatments (PT)				Constant treatments (CT)	
Model	Calibration data	Tr A 1(14-day)	Tr B 2(14-day)	Tr A 1(21-day)	Tr B 2(21-day)	Tr C(14-day)	Tr C(21-day)
IT	PT alone/CT alone	5.0	3.5	3.4	3.9	5.4	2.6
	All data	5.4	3.1	3.3	3.8	5.4	4.7
SD	PT alone/CT alone	8.3	7.2	23.6	13.4	5.7	4.0
	All data	7.3	8.3	22.7	13.4	5.1	5.0

1 Pulsed treatment with a short interval in uncontaminated water between imidacloprid pulses.

2 Pulsed treatment with a long interval in uncontaminated water between imidacloprid pulses.

**Figure 4:**
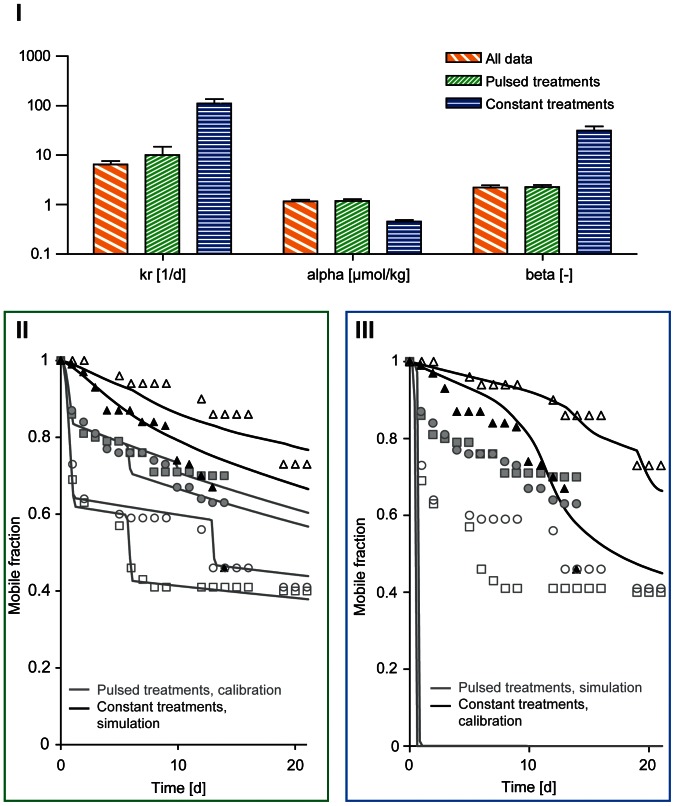
Parameter estimates and fraction of mobile animals simulated with the individual tolerance distribution model. Parameter estimates of individual tolerance models calibrated with all data, pulsed data only, and constant treatments only (I). Calibration to pulsed treatments (gray lines and gray symbols) and using these parameters to simulate the fraction of mobile animals in the constant scenario (black lines) are shown in the green box (II). Vice versa, calibration to constant treatments (black lines and black symbols) and using these parameters to simulate the fraction of mobile animals in the pulsed scenario (grey lines) are shown in the blue box (III). Symbols represent the data: black triangles are the mobile fraction in constant treatments (C), gray squares are data from pulsed treatments A and gray circles are from pulsed treatments B. Closed symbols are data from 14-day experiment and open symbols from 21-day experiment.

Parameters differed when the models were calibrated with either pulsed or constant survival/immobility data. In Figure 4, parameter estimates of the damage recovery rate (*k*_r_), the median of the threshold distribution (*alpha*), and the width of the distribution (*beta*) for pulsed treatments, constant treatments and all data are illustrated. The biggest differences between constant and pulsed calibration data can be seen in the values of the parameter *beta*, which was extremely high for the constant data compared to the pulsed data. The value of *beta* determines the width of the threshold distribution – the higher the value is, the narrower is the distribution and the steeper is the drop in the fraction of survival/mobility close to the median value *alpha*. This would imply that the individuals in a population react fairly similarly to starvation, or the damage describing decreasing lipid content.

However, parameter estimates should not be over-interpreted because they are linked to each other (parameter co-variation). To compare among calibration data sets in a more reliable manner, the whole set of parameters can be used to simulate the survival in another exposure type (i.e. from calibration to the constant data set to simulate survival in the pulsed scenario and vice versa). When the pulsed calibration data was used, mobility in the constant scenario was rather well predicted with MPE of 5.5±9.5% (14-day experiment) and 4.4±2.4% (21-day experiment) (Figure 4, II). However, the high mortality in the end of constant treatments was not captured (i.e. the effect of starvation). When the constant calibration data was used, the mobility in the pulsed scenario could not be predicted well; the mobile fraction went directly to zero in all pulsed treatments (Figure 4, III).

This simulation result indicates that there are different processes, described by very different model parameters, governing the mobility under long, constant exposure to imidacloprid vs. the pulsed exposure scenario. The toxic processes in the constant treatments can be related to a low degree of imidacloprid binding in nicotinic receptors making organisms passive and causing death via impaired movements and starvation. In the pulsed treatments other toxic effects from higher degree of binding to the target sites play also a role. This can be seen also as a difference between the adverse effects of imidacloprid in pulsed treatments, where imidacloprid immobilizes the organisms immediately, and more chronic effects like starvation which appears as a result from impaired movements in long constant exposure. However, we cannot rule out the possibility that the differences in model parameters can also be caused by differing proportions of dead and immobile individuals among treatment types (the model treats them equally, because immobile individuals were removed). Almost only mortality was observed in the constant treatments while there were much more immobile individuals than dead ones in the pulsed treatments (pie charts in Figures 1 and 2, part III).

### Mortality in the Starvation Experiment

**Figure 5:**
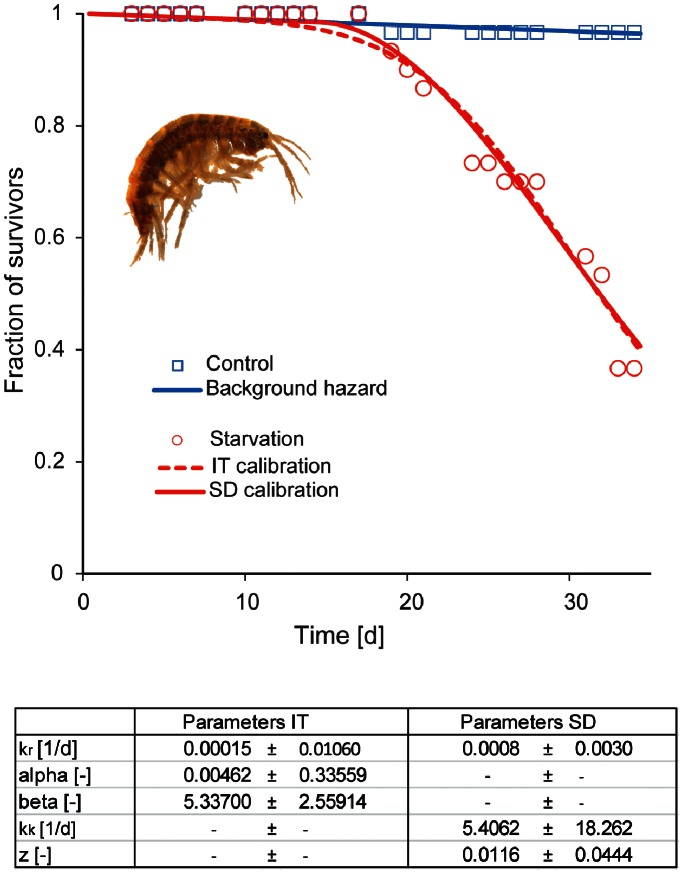
Calibration of the starvation model. The table shows the calibrated parameter values and their standard deviation.

**Figure 6:**
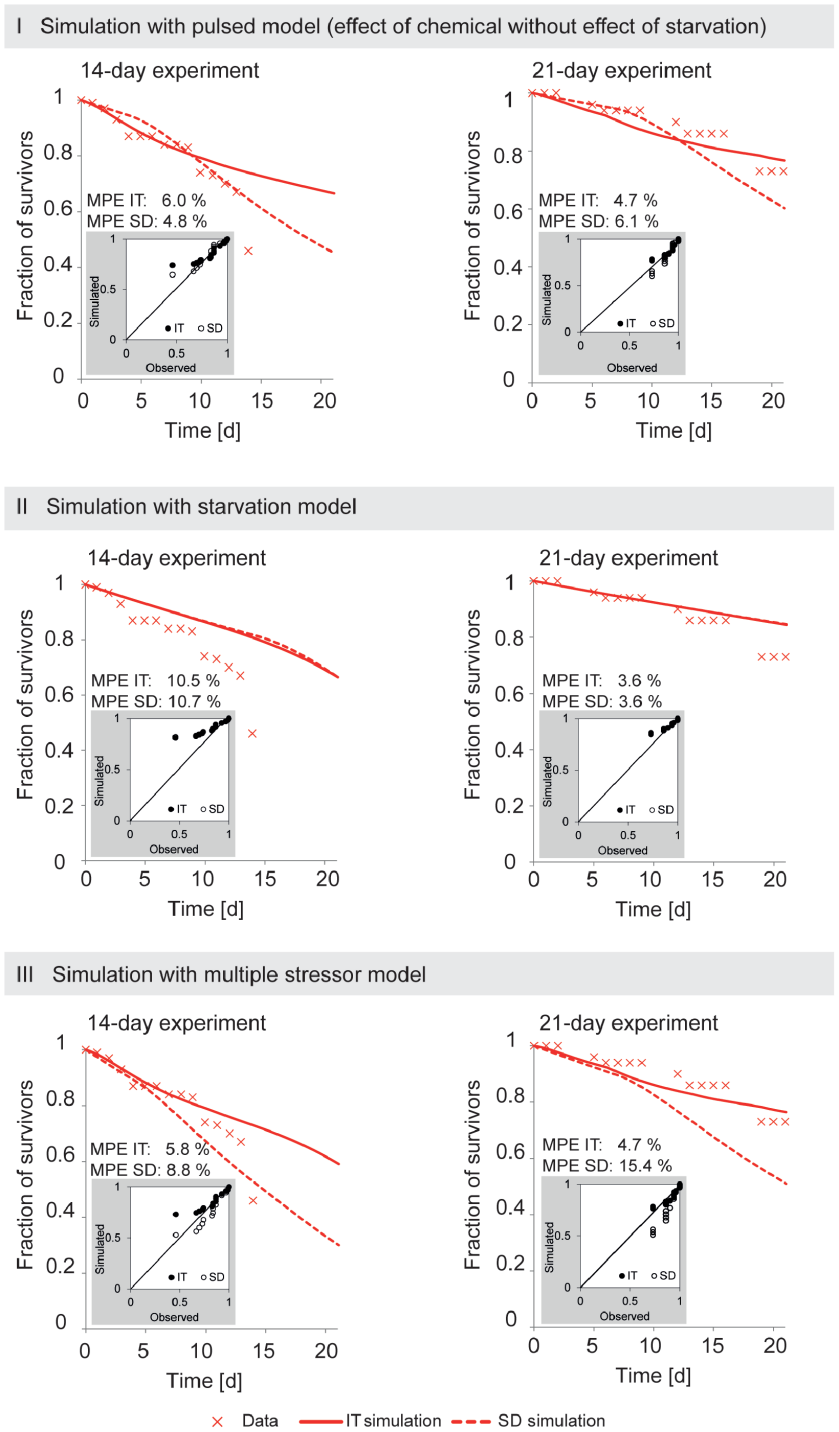
Simulation of survival of ***Gammarus pulex*** **in constant imidacloprid exposure according to the chemical stress model (I), starvation model (II), and multiple stressor model (III).** In the starvation model (II), lack of food (*LF*) for the 14-day experiment was set to 1.0 and for the 21-day experiment 0.5 due to differences in feeding activity (no feeding in 14-day experiment, ca. 50% reduced feeding in the 21-day experiment). The chemical stress model was GUTS calibrated with pulsed toxicity data sets.

In one of the target organisms, the tobacco whitefly (*Bemisia tabaci*), imidacloprid has been shown to cause starvation [41], [42]. Even though the exposure route is partly different for aquatic species (oral uptake alone for the tobacco whitefly versus oral uptake and diffusion from water for *G. pulex*), low concentrations of imidacloprid can cause death of non-target amphipods in aquatic environments via behavioral changes which prevent the organisms from feeding. To investigate whether other effects of imidacloprid than starvation play a role in the constant exposures, we performed a pure starvation experiment where no imidacloprid was added. We calibrated the TKTD models with food limitation as dose metric and the models were then used to simulate mortality in the constant imidacloprid treatments. Results of the starvation experiment and calibration of the models are shown in Figure 5. The simulation of survival in the constant lack of food conditions, representing our constant imidacloprid exposure, showed rather poor agreement with the measured values, especially for the 14-day experiment (Figure 6, part II). However it must be noted, that this prediction is based only on starvation stress. As cholinergic neurotransmission has been suspected to have a central role in neurotransmission in invertebrate central nervous system [43], likely also other pathways of toxicity, e.g. failure of respiration, than starvation through impaired movements occur in imidacloprid exposure.

### Survival in Multiple Stress Conditions

We developed a multiple stress model, combining the effects of starvation and the other toxic pathways of the chemical, for simulating survival in the constant imidacloprid exposure. The SD model for multiple stressors did not predict survival well when we compare mean percentage error (MPE) values between three different model types: The 14-day experiment was better predicted by the chemical stress model and the 21-day experiment by the starvation model (Figure 6). However, as discussed above, the IT model had a better fit to the data (Figure 2, Table 2) and therefore we should focus on comparing results given by this model (see solid line in Figure 6). The agreement of the simulation with the data increased with the multiple stress model, when compared to simulations using either of the individual stressor models (i.e. chemical stress and starvation) alone especially in the case of the 14-day experiment (Figure 6). Using the multiple stress model did improve the predictive power, however, one pattern in the data, i.e. the sudden drop in survival at the end of the experiments, was not predicted quantitatively.

### Conclusions

Organisms in nature are facing multiple stress conditions, natural and anthropogenic. We have investigated the effects of the insecticide imidacloprid in *Gammarus pulex* and showed that multiple stress pathways might influence organism survival, rather than just a single mechanism of toxicity or stress pathway alone. By binding to acetylcholine receptors, imidacloprid impairs invertebrate movements and feeding behavior and therefore, in addition to other pathways of toxicity, starvation can influence organism survival if the exposure lasts longer than the duration of standard toxicity tests. Another noteworthy point of our findings is that the effect of reduced feeding on invertebrate survival is affected by food availability and initial nutritional status of organisms which vary along with the season. Nevertheless, in aquatic systems the exposure times are usually short due to water flow and dilution. We showed that in these conditions, organism movements and feeding can recover fast after the exposure and therefore the stress from starvation is reduced, however, other toxic pathways can still affect organism survival.

## Supporting Information

Figure S1 **Lipid content [%] of** ***Gammarus pulex*** **at the end of the second experiment in control and treatments A, B and C.** Green color of the box denotes pulsed treatments (Tr A and B) and red color constant treatment (Tr C). The numbers are the median values represented by the black line in boxes.
(TIF)

File S1 **Supporting Tables. Table S1.** Composition of artificial pond water (APW) and stock solutions. **Table S2.** Imidacloprid concentrations in water in 14-day experiment. **Table S3.** Imidacloprid concentrations in water in 21-day experiment. **Table S4.** Water characteristics in 14-day experiment. **Table S5.** Water characteristics in 21-day experiment. **Table S6.** Data on internal concentrations in 14-day experiment: Tr A. **Table S7.** Data on internal concentrations in 14-day experiment: Tr B. **Table S8.** Data on internal concentrations in 14-day experiment: Tr C. **Table S9.** Cumulative food consumption (leaf discs/*G. pulex* individual) in 14-day experiment. Value is corrected with number of mobile individuals in the beaker. **Table S10.** Cumulative food consumption (mg/*G. pulex* individual) in 21-day experiment. Nm denotes “not measured” in that time point. This is taken into account in the value in the next time point. **Table S11.** Lipid content of immobile *Gammarus pulex* individuals in the 21-day experiment and mobile individuals in the end of experiment. **Table S12.** Number of mobile and immobile individuals in the 14-day experiment. Cint A and Cint B denotes beakers which were used to sample mobile individuals and were not used for survival modeling. **Table S13.** Number of mobile and immobile individuals in the 21-day experiment. **Table S14.** Parameter estimates for different calibration data sets. Parameter estimates of stochastic death (SD) and individual tolerance distribution (IT) models based on fit using log-likelihood function or ordinary least squares fit.
(DOCX)

## References

[1] Pimentel D (2009) Pesticides and pest control. In: Peshin R, Dhawan A, editors. Integrated pest management: Innovation-development process: Springer Science+Business Media.

[2] Jeschke P, Nauen R, Schindler M, Elbert A (2010) Overview of the status and global strategy for neonicotinoids. Journal of Agricultural and Food Chemistry 59: 2897–2908. 20565065 10.1021/jf101303g

[3] Jeschke P, Nauen R (2008) Nervous System. In: Krämer W, Schirmer U, editors. Modern Crop Protection Compounds. Wenheim, Germany: Wiley-VCH Verlag GmbH. 927–1088.

[4] Abbink J (1991) The biochemistry of imidacloprid. Pflanzenschutz-Nachrichten Bayer 42: 183–195.

[5] Tomizawa M, Casida JE (2003) Selective toxicity of neonicotinoids attributable to specificity of insect and mammalian nicotinic receptors. Annual review of entomology 48: 339–364. 10.1146/annurev.ento.48.091801.112731 12208819

[6] Starner K, Goh K (2012) Detections of the neonicotinoid insecticide imidacloprid in surface waters of three agricultural regions of California, USA, 2010–2011. Bulletin of Environmental Contamination and Toxicology 88: 316–321. 22228315 10.1007/s00128-011-0515-5

[7] Kreuger J, Graaf S, Patring J, Adielsson S (2010) Pesticides in surface water in areas with open ground and greenhouse horticultural crops in Sweden 2008. Uppsala: Swedish University of Agricultural Sciences.

[8] Beketov M, Liess M (2008) Potential of 11 pesticides to initiate downstream drift of stream macroinvertebrates. Archives of Environmental Contamination and Toxicology 55: 247–253. 18180861 10.1007/s00244-007-9104-3

[9] Kreuger J (1998) Pesticides in stream water within an agricultural catchment in southern Sweden, 1990–1996. Science of The Total Environment 216: 227–251. 9646531 10.1016/s0048-9697(98)00155-7

[10] Wittmer IK, Bader HP, Scheidegger R, Singer H, Lück A, et al (2010) Significance of urban and agricultural land use for biocide and pesticide dynamics in surface waters. Water Research 44: 2850–2862. 20188390 10.1016/j.watres.2010.01.030

[11] Lind RJ, Clough MS, Reynolds SE, Earley FGP (1998) [3H]Imidacloprid labels high- and low-affinity nicotinic acetylcholine receptor-like binding sites in the aphid *Myzus persicae* (Hemiptera: Aphididae). Pesticide Biochemistry and Physiology 62: 3–14.

[12] Ashauer R, Caravatti I, Hintermeister A, Escher BI (2010) Bioaccumulation kinetics of organic xenobiotic pollutants in the freshwater invertebrate *Gammarus pulex* modeled with prediction intervals. Environmental Toxicology and Chemistry 29: 1625–1636. 20821614 10.1002/etc.175

[13] Ashauer R, Hintermeister A, O’Connor I, Elumelu M, Hollender J, et al (2012) Significance of xenobiotic metabolism for bioaccumulation kinetics of organic chemicals in *Gammarus pulex* . Environmental Science & Technology 46: 3498–3508. 22321051 10.1021/es204611h PMC3308200

[14] Mohr S, Berghahn R, Schmiediche R, Hübner V, Loth S, et al (2012) Macroinvertebrate community response to repeated short-term pulses of the insecticide imidacloprid. Aquatic Toxicology 110–111: 25–36. 10.1016/j.aquatox.2011.11.016 22252165

[15] Berghahn R, Mohr S, Hübner V, Schmiediche R, Schmiedling I, et al (2012) Effects of repeated insecticide pulses on macroinvertebrate drift in indoor stream mesocosms. Aquatic Toxicology 122–123: 56–66. 10.1016/j.aquatox.2012.05.012 22721787

[16] Kreutzweiser DP, Thompson DG, Scarr TA (2009) Imidacloprid in leaves from systemically treated trees may inhibit litter breakdown by non-target invertebrates. Ecotoxicology and Environmental Safety 72: 1053–1057. 18973940 10.1016/j.ecoenv.2008.09.017

[17] Alexander AC, Culp JM, Liber K, Cessna AJ (2007) Effects of insecticide exposure on feeding inhibition in mayflies and oligochaetes. Environmental Toxicology and Chemistry 26: 1726–1732. 17702348 10.1897/07-015r.1

[18] Azevedo-Pereira HVS, Lemos ML, Soares AVM (2011) Behaviour and growth of *Chironomus riparius* Meigen (Diptera: Chironomidae) under imidacloprid pulse and constant exposure scenarios. Water, Air, & Soil Pollution 219: 215–224.

[19] Anderson N (1979) Detritus processing by macroinvertebrates in stream ecosystems. Annual review of entomology 24: 351–377.

[20] Naylor C, Maltby L, Calow P (1989) Scope for growth in *Gammarus pulex*, a freshwater benthic detritivore. Hydrobiologia 188–189: 517–523.

[21] Moza PN, Hustert K, Feicht E, Kettrup A (1998) Photolysis of imidacloprid in aqueous solution. Chemosphere 36: 497–502.

[22] Zheng W, Liu W-p, Wen Y-z, Lee S-J (2004) Photochemistry of insecticide imidacloprid: direct and sensitized photolysis in aqueous medium. Journal of Environmental Sciences 16: 539–542. 15495951

[23] Kretschmann A, Ashauer R, Preuss TG, Spaak P, Escher BI, et al (2011) Toxicokinetic model describing bioconcentration and biotransformation of diazinon in *Daphnia magna* . Environmental Science & Technology 45: 4995–5002. 21561125 10.1021/es104324v

[24] Jager T, Albert C, Preuss TG, Ashauer R (2011) General unified threshold model of survival - a toxicokinetic-toxicodynamic framework for ecotoxicology. Environmental Science & Technology 45: 2529–2540. 21366215 10.1021/es103092a

[25] Nyman A-M, Schirmer K, Ashauer R (2012) Toxicokinetic-toxicodynamic modelling of survival of *Gammarus pulex* in multiple pulse exposures to propiconazole: model assumptions, calibration data requirements and predictive power. Ecotoxicology - *in press*. 10.1007/s10646-012-0917-0 PMC3431474 22562719

[26] Motulsky HJ, Christopoulos A (2003) Fitting models to biological data using linear and nonlinear regression. A practical guide to curve fitting. San Diego CA, USA: GraphPad Software Inc., www.graphpad.com.

[27] Ramakrishnan R, Suiter DR, Nakatsu CH, Bennett GW (2000) Feeding inhibition and mortality in *Reticulitermes flavipes* (Isoptera: Rhinotermitidae) after exposure to imidacloprid-treated soils. Journal of Economic Entomology 93: 422–428. 10826195 10.1603/0022-0493-93.2.422

[28] Blažič M, Trebbˇse P, Drobne D (2005) Effect of imidacloprid on growth, feeding rate and activity of ache and GST enzymes in the terrestrial isopods *Porcellio scaber* (Isopoda, Crustacea). Lectures and papers presented at the 7th Slovenian Conference on Plant Protection. Zreče, Slovenia. 106–113 (Abstract).

[29] Drobne D, Blažič M, Van Gestel CAM, Lešer V, Zidar P, et al (2008) Toxicity of imidacloprid to the terrestrial isopod *Porcellio scaber* (Isopoda, Crustacea). Chemosphere 71: 1326–1334. 18190949 10.1016/j.chemosphere.2007.11.042

[30] Lind RJ, Hardick DJ, Blagbrough IS, Potter BVL, Wolstenholme AJ, et al (2001) [3H]-Methyllycaconitine: a high affinity radioligand that labels invertebrate nicotinic acetylcholine receptors. Insect Biochemistry and Molecular Biology 31: 533–542. 11267892 10.1016/s0965-1748(00)00153-3

[31] Wellmann H, Gomes M, Lee C, Kayser H (2004) Comparative analysis of neonicotinoid binding to insect membranes: II. An unusual high affinity site for [3H]thiamethoxam in *Myzus persicae* and *Aphis craccivora* . Pest Management Science 60: 959–970. 15481822 10.1002/ps.920

[32] Stoughton S, Liber K, Culp J, Cessna A (2008) Acute and chronic toxicity of imidacloprid to the aquatic invertebrates *Chironomus tentans* and *Hyalella azteca* under constant- and pulse-exposure conditions. Archives of Environmental Contamination and Toxicology 54: 662–673. 18214581 10.1007/s00244-007-9073-6

[33] Ashauer R, Boxall ABA, Brown CD (2007) Simulating toxicity of carbaryl to *Gammarus pulex* after sequential pulsed exposure. Environmental Science & Technology 41: 5528–5534. 17822128 10.1021/es062977v

[34] Ashauer R, Hintermeister A, Caravatti I, Kretschmann A, Escher BI (2010) Toxicokinetic and toxicodynamic modeling explains carry-over toxicity from exposure to diazinon by slow organism recovery. Environmental Science & Technology 44: 3963–3971. 20397634 10.1021/es903478b

[35] Sroda S, Cossu-Leguille C (2011) Seasonal variability of antioxidant biomarkers and energy reserves in the freshwater gammarid *Gammarus roeseli* . Chemosphere 83: 538–544. 21215985 10.1016/j.chemosphere.2010.12.023

[36] Krog J (1954) The influence of seasonal environmental changes upon the metabolism, lethal temperature and rate of heart beat of *Gammarus limnaeus* (Smith) taken from an Alaskan Lake Biological Bulletin. 107: 397–410.

[37] Böttger R, Schaller J, Mohr S (2012) Closer to reality – the influence of toxicity test modifications on the sensitivity of *Gammarus roeseli* to the insecticide imidacloprid. Ecotoxicology and Environmental Safety 81: 49–54. 22575057 10.1016/j.ecoenv.2012.04.015

[38] Newman MC, McCloskey JT (2000) The individual tolerance concept is not the sole explanation for the probit dose-effect model. Environmental Toxicology and Chemistry 19: 520–526.

[39] Bliss CI (1935) The calculation of the dosage-mortality curve. Annals of Applied Biology 22: 134–167.

[40] Dauterman WC (1994) Adaptation to toxicants. In: Hodson E, Levi P, editors. Introduction to biochemical toxicology. Norwalk, CT, USA: Apleton and Lange.

[41] Nauen R (1995) Behaviour modifying effects of low systemic concentrations of imidacloprid on *Myzus persicae* with special reference to an antifeeding response. Pesticide Science 44: 145–153.

[42] Nauen R, Koob B, Elbert A (1998) Antifeedant effects of sublethal dosages of imidacloprid on *Bemisia tabaci* . Entomologia Experimentalis et Applicata 88: 287–293.

[43] Florey E (1963) Acetylcholine in invertebrate nervous system. Canadian Journal of Biochemistry and Physiology 41: 2619–2626. 14099718

