# Supplementary figures and images for: The Insecticide Imidacloprid Causes Mortality of the Freshwater Amphipod Gammarus pulex by Interfering with Feeding Behavior
Source: PLoS One. 2013 May 15;8(5):e62472. doi: 10.1371/journal.pone.0062472 (PMC3655172; doi:10.1371/journal.pone.0062472)

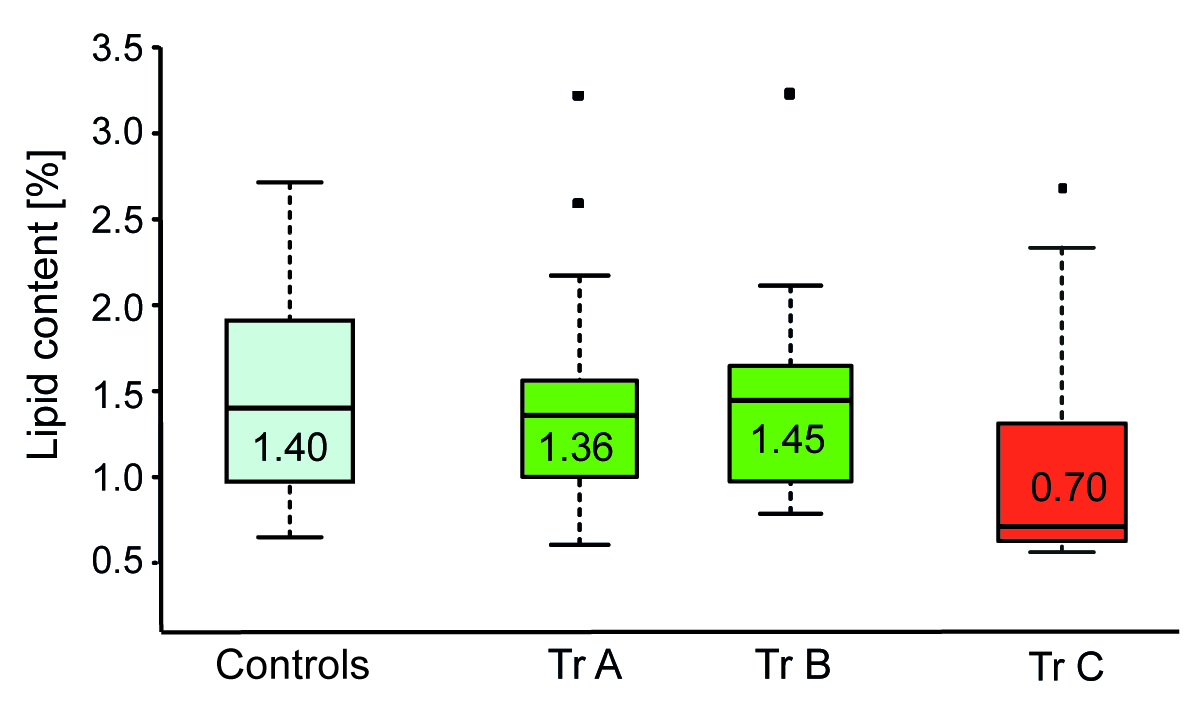

Supplement: Figure S1 — Lipid content [%] of Gammarus pulex at the end of the second experiment in control and treatments A, B and C. Green color of the box denotes pulsed treatments (Tr A and B) and red color constant treatment (Tr C). The numbers are the median values represented by the black line in boxes. (TIF) [file pone.0062472.s001.tif]
