# Supplementary material for: The Insecticide Imidacloprid Causes Mortality of the Freshwater Amphipod Gammarus pulex by Interfering with Feeding Behavior
Source: PLoS One. 2013 May 15;8(5):e62472. doi: 10.1371/journal.pone.0062472 (PMC3655172; doi:10.1371/journal.pone.0062472)
Supplement: File S1 — Supporting Tables. Table S1. Composition of artificial pond water (APW) and stock solutions. Table S2. Imidacloprid concentrations in water in 14-day experiment. Table S3. Imidacloprid concentrations in water in 21-day experiment. Table S4. Water characteristics in 14-day experiment. Table S5. Water characteristics in 21-day experiment. Table S6. Data on internal concentrations in 14-day experiment: Tr A. Table S7. Data on internal concentrations in 14-day experiment: Tr B. Table S8. Data on internal concentrations in 14-day experiment: Tr C. Table S9. Cumulative food consumption (leaf discs/G. pulex individual) in 14-day experiment. Value is corrected with number of mobile individuals in the beaker. Table S10. Cumulative food consumption (mg/G. pulex individual) in 21-day experiment. Nm denotes “not measured” in that time point. This is taken into account in the value in the next time point. Table S11. Lipid content of immobile Gammarus pulex individuals in the 21-day experiment and mobile individuals in the end of experiment. Table S12. Number of mobile and immobile individuals in the 14-day experiment. Cint A and Cint B denotes beakers which were used to sample mobile individuals and were not used for survival modeling. Table S13. Number of mobile and immobile individuals in the 21-day experiment. Table S14. Parameter estimates for different calibration data sets. Parameter estimates of stochastic death (SD) and individual tolerance distribution (IT) models based on fit using log-likelihood function or ordinary least squares fit. (DOCX) [file pone.0062472.s002.docx]

*Supporting Information*

Low concentrations of the pesticide imidacloprid cause mortality of freshwater amphipod *Gammarus pulex* by interfering with feeding behavior

Anna-Maija Nyman ^a,b^, Anita Hintermeister ^a^, Kristin Schirmer ^a, b, c^, Roman Ashauer ^a^

^a^ Department of Environmental Toxicology, Eawag - Swiss Federal Institute of Aquatic Science and Technology, Dübendorf, Switzerland

^b^ Department of Environmental Systems Science, ETH Zürich, Zürich, Switzerland

^c^ School of Architecture, Civil and Environmental Engineering, EPF Lausanne, Lausanne, Switzerland

^d^ Environment Department, University of York, Heslington, York, UK

Contents

[1. Artificial pond water 2](#_Toc347493745)

[2. Measured exposure concentrations 2](#_Toc347493746)

[3. pH, conductivity and oxygen concentrations in water 3](#_Toc347493747)

[4. Raw data on chemical internal concentrations in experiment 1 5](#_Toc347493748)

[5. Raw data on feeding activity 8](#_Toc347493749)

[6. Lipid content of *G. pulex* in the end of experiment 2 10](#_Toc347493750)

[7. Data on mobile fractions 15](#_Toc347493751)

[8. Fitting survival models with likelihood function 17](#_Toc347493752)

# Artificial pond water

**Table S1. Composition of artificial pond water (APW) and stock solutions.**

|  | Concentration in stock | Concentration in APW |
| --- | --- | --- |
| CaCl_2_^.^ 2H_2_O (calcium chloride) | 58.80 g/L distilled water | 294.0 mg/L |
| MgSO_4_ ^.^ 7H_2_O (magnesium sulphate) | 24.65 g/L distilled water | 123.3 mg/L |
| NaHCO_3_ (sodium hydrogen carbonate) | 12.95 g/L distilled water | 64.8 mg/L |
| KCl (potassium chloride) | 1.15 g/L distilled water | 5.8 mg/L |

Artificial pond water for experiments were prepared by adding 100 ml of each stock to 20 L of nanopure water [[1](#_ENREF_1)].

# Measured exposure concentrations

**Table S2. Imidacloprid concentrations in water in 14-day experiment.**

| **Time** | **Tr. A** | **Tr. B** | **Tr. C** |  |
| --- | --- | --- | --- | --- |
| **[d]** | **[µmol/L]** | **[µmol/L]** | **[µmol/L]** |  |
| 0.00 | 0.3595 | 0.3594 | 0.0638 |  |
| 0.94 | 0.3539 | 0.3532 | 0.0625 |  |
| 0.95 | 0.0000 | 0.0000 | 0.0625 | inserted |
| 1.94 | 0.0014 | 0.0008 | 0.0623 |  |
| 2.94 | 0.0007 | 0.0005 | 0.0616 |  |
| 3.94 | 0.0009 | 0.0005 | 0.0619 |  |
| 4.94 | 0.0008 | 0.0005 | 0.0616 |  |
| 4.98 | 0.3487 | 0.0000 | 0.0627 |  |
| 5.90 | 0.3443 | 0.0006 | 0.0620 |  |
| 5.91 | 0.0000 | 0.0000 | 0.0620 | inserted |
| 6.94 | 0.0007 | 0.0000 | 0.0617 |  |
| 7.94 | 0.0010 | 0.0000 | 0.0614 |  |
| 8.92 | 0.0009 | 0.0003 | 0.0613 |  |
| 8.98 | 0.0000 | 0.3531 | 0.0600 |  |
| 9.92 | 0.0010 | 0.3420 | 0.0611 |  |
| 9.93 | 0.0000 | 0.0000 | 0.0611 | inserted |
| 10.96 | 0.0009 | 0.0000 | 0.0632 |  |
| 11.98 | 0.0000 | 0.0007 | 0.0626 |  |
| 12.94 | 0.0000 | 0.0006 | 0.0618 |  |
| 13.94 | 0.0000 | 0.0006 | 0.0614 |  |

**Table S3. Imidacloprid concentrations in water in 21-day experiment.**

| **Time** | **Tr. A** | **Tr. B** | **Tr. C** |  |
| --- | --- | --- | --- | --- |
| **[d]** | **[µmol/L]** | **[µmol/L]** | **[µmol/L]** |  |
| 0 | 0.5517 | 0.5605 | 0.0480 |  |
| 1.021 | 0.5406 | 0.5353 | 0.0480 |  |
| 1.1 | 0.0000 | 0.0000 | 0.0480 | inserted |
| 5.000 | 0.0004 | 0.0001 | 0.0480 |  |
| 5.007 | 0.6365 | 0.0001 | 0.0480 |  |
| 6.007 | 0.6228 | 0.0001 | 0.0427 |  |
| 6.01 | 0.0000 | 0.0001 | 0.0606 | inserted |
| 9.017 | 0.0003 | 0.0001 | 0.0590 |  |
| 12.013 | 0.0003 | 0.0002 | 0.0590 |  |
| 12.020 | 0.0003 | 0.6590 | 0.0590 |  |
| 13.024 | 0.0003 | 0.6389 | 0.0579 |  |
| 13.100 | 0.0003 | 0.0000 | 0.0617 | inserted |
| 16.017 | 0.0001 | 0.0004 | 0.0583 |  |
| 18.997 | 0.0001 | 0.0004 | 0.0585 |  |
| 19.015 | 0.0001 | 0.0004 | 0.0642 |  |
| 21.115 | 0.0001 | 0.0001 | 0.0596 |  |

# pH, conductivity and oxygen concentrations in water

**Table S4. Water characteristics in 14-day experiment.**

| **Beaker** | **Date** | **pH** | **Conductivity [µS/cm]** | **Oxygen [mg/L]** |
| --- | --- | --- | --- | --- |
| Control A | 23.11.2010 | 7.07 | 602 | 2.48 |
| Solvent control A | 23.11.2010 | 7.00 | 601 | 2.87 |
| A3 | 23.11.2010 | 6.78 | 601 | 2.34 |
| Control B | 23.11.2010 | 7.02 | 607 | 2.67 |
| Solvent control B | 23.11.2010 | 6.90 | 600 | 4.00 |
| B3 | 23.11.2010 | 6.86 | 597 | 3.15 |
| Control C | 23.11.2010 | 6.86 | 592 | 2.35 |
| Solvent control C | 23.11.2010 | 6.80 | 598 | 2.16 |
| C4 | 23.11.2010 | 6.90 | 600 | 2.90 |
| Control A | 01.12.2010 | 6.97 | 597 | 4.17 |
| Solvent control A | 01.12.2010 | 6.80 | 592 | 3.91 |
| A3 | 01.12.2010 | 6.78 | 585 | 4.32 |
| Control B | 01.12.2010 | 6.83 | 599 | 3.91 |
| Solvent control B | 01.12.2010 | 6.91 | 603 | 2.95 |
| B3 | 01.12.2010 | 6.88 | 590 | 4.32 |
| Control C | 01.12.2010 | 6.76 | 597 | 2.83 |
| Solvent control C | 01.12.2010 | 6.79 | 606 | 2.44 |
| C4 | 01.12.2010 | 6.87 | 597 | 3.24 |
| Control A | 06.12.2010 | 7.24 | 620 | 3.39 |
| Solvent control A | 06.12.2010 | 7.10 | 605 | 4.26 |
| A3 | 06.12.2010 | 7.04 | 613 | 3.26 |
| Control B | 06.12.2010 | 7.10 | 622 | 3.45 |
| Solvent control B | 06.12.2010 | 7.03 | 630 | 2.63 |
| B3 | 06.12.2010 | 7.26 | 616 | 3.25 |
| Control C | 06.12.2010 | 6.97 | 602 | 2.37 |
| Solvent control C | 06.12.2010 | 6.93 | 619 | 2.20 |
| C4 | 06.12.2010 | 6.89 | 607 | 1.39 |

**Table S5. Water characteristics in 21-day experiment.**

| **Beaker** | **Date** | **pH** | **Conductivity [µS/cm]** | **Oxygen [mg/L]** |
| --- | --- | --- | --- | --- |
| Control A | 24.02.2011 | 7.20 | 591 | 6.96 |
| Solvent control A | 24.02.2011 | 7.33 | 603 | 6.10 |
| Control B | 24.02.2011 | 7.48 | 601 | 6.55 |
| Solvent control B | 24.02.2011 | 7.32 | 603 | 6.90 |
| Control A | 24.02.2011 | 6.93 | 602 | 4.77 |
| Solvent control A | 24.02.2011 | 7.00 | 611 | 4.55 |
| Control B | 24.02.2011 | 7.14 | 601 | 4.90 |
| Solvent control B | 24.02.2011 | 6.99 | 596 | 5.01 |
| Control C | 24.02.2011 | 7.02 | 599 | 4.78 |
| Solvent control C | 24.02.2011 | 7.00 | 599 | 4.63 |
| A7 | 24.02.2011 | 7.36 | 593 | 6.90 |
| A4 | 24.02.2011 | 7.39 | 603 | 7.21 |
| A1 | 24.02.2011 | 7.42 | 601 | 7.07 |
| B7 | 24.02.2011 | 7.44 | 600 | 6.70 |
| B4 | 24.02.2011 | 7.46 | 595 | 7.17 |
| B1 | 24.02.2011 | 7.44 | 606 | 6.80 |
| A7 | 24.02.2011 | 7.13 | 624 | 4.14 |
| A4 | 24.02.2011 | 7.09 | 604 | 4.54 |
| A1 | 24.02.2011 | 7.13 | 604 | 4.60 |
| B7 | 24.02.2011 | 7.05 | 599 | 4.62 |
| B4 | 24.02.2011 | 7.07 | 596 | 4.99 |
| B1 | 24.02.2011 | 7.04 | 601 | 5.01 |
| C7 | 24.02.2011 | 7.05 | 600 | 5.07 |
| C4 | 24.02.2011 | 7.06 | 601 | 5.08 |
| C1 | 24.02.2011 | 7.01 | 595 | 4.27 |
| Control A | 01.03.2011 | 7.67 | 589 | 6.97 |
| Solvent control A | 01.03.2011 | 7.71 | 599 | 6.52 |
| Control A | 01.03.2011 | 7.11 | 612 | 3.76 |
| Solvent control A | 01.03.2011 | 7.12 | 609 | 4.22 |
| A7 | 01.03.2011 | 7.71 | 595 | 6.93 |
| A4 | 01.03.2011 | 7.74 | 607 | 6.47 |
| A1 | 01.03.2011 | 7.75 | 602 | 7.13 |
| A7 | 01.03.2011 | 7.38 | 611 | 5.75 |
| A4 | 01.03.2011 | 7.40 | 601 | 5.11 |
| A1 | 01.03.2011 | 7.31 | 606 | 5.72 |
| Control B | 08.03.2011 | 7.65 | 587 | 7.89 |
| Solvent control B | 08.03.2011 | 7.71 | 595 | 7.57 |
| Control B | 08.03.2011 | 7.02 | 608 | 5.13 |
| Solvent control B | 08.03.2011 | 7.13 | 608 | 5.24 |
| B7 | 08.03.2011 | 7.78 | 595 | 7.20 |
| B4 | 08.03.2011 | 7.82 | 594 | 7.46 |
| B1 | 08.03.2011 | 7.79 | 597 | 7.92 |
| B7 | 08.03.2011 | 7.17 | 618 | 5.58 |
| B4 | 08.03.2011 | 7.22 | 604 | 6.22 |
| B1 | 08.03.2011 | 7.18 | 611 | 5.96 |
| Control C | 14.03.2011 | 7.77 | 583 | 5.42 |
| Solvent control C | 14.03.2011 | 7.75 | 585 | 5.63 |
| Control C | 14.03.2011 | 7.33 | 598 | 4.80 |
| Solvent control C | 14.03.2011 | 7.12 | 596 | 4.09 |
| C7 | 14.03.2011 | 7.67 | 585 | 5.04 |
| C4 | 14.03.2011 | 7.72 | 590 | 5.19 |
| C1 | 14.03.2011 | 7.74 | 587 | 4.94 |
| C7 | 14.03.2011 | 6.95 | 601 | 2.64 |
| C4 | 14.03.2011 | 7.02 | 593 | 2.88 |
| C1 | 14.03.2011 | 7.01 | 593 | 3.33 |

# Raw data on chemical internal concentrations in experiment 1

**Table S6. Data on internal concentrations in 14-day experiment: Tr A**

| **Time [d]** | **mobile/immobile** | **Cint [µmol/kg]** |
| --- | --- | --- |
| 0.96 | immobile | 0.813208 |
| 0.96 | immobile | 2.016268 |
| 0.96 | immobile | 0.977897 |
| 0.96 | immobile | 2.607686 |
| 1.96 | immobile | 0.33252 |
| 1.96 | immobile | 0.412659 |
| 1.96 | immobile | 0.423959 |
| 7.96 | immobile | 0.313245 |
|  |  |  |
| 0.96 | mobile | 0.832844 |
| 0.96 | mobile | 0.652293 |
| 0.96 | mobile | 1.001624 |
| 4.96 | mobile | 0.167125 |
| 4.96 | mobile | 0.163975 |
| 4.96 | mobile | 0.034461 |
| 5.96 | mobile | 0.604519 |
| 5.96 | mobile | 0.92591 |
| 5.96 | mobile | 1.421349 |
| 13.96 | mobile | 0.15957 |
| 13.96 | mobile | 0.165935 |
| 13.96 | mobile | 0.131966 |
| 13.96 | mobile | 0.112992 |
| 13.96 | mobile | 0.120999 |
| 13.96 | mobile | 0.03089 |
| 13.96 | mobile | 0.105832 |
| 13.96 | mobile | 0.1696 |
| 13.96 | mobile | 0.130184 |
| 13.96 | mobile | 0.114761 |
| 13.96 | mobile | 0.096588 |
| 13.96 | mobile | 0.170583 |
| 13.96 | mobile | 0.122233 |
| 13.96 | mobile | 0.176417 |
| 13.96 | mobile | 0.194873 |
| 13.96 | mobile | 0.128837 |
| 13.96 | mobile | 0.081094 |
| 13.96 | mobile | 0.131151 |
| 13.96 | mobile | 0.03709 |
| 13.96 | mobile | 0.025169 |
| 13.96 | mobile | 0.124625 |

**Table S7. Data on internal concentrations in 14-day experiment: Tr B**

| **Time [d]** | **mobile/immobile** | **Cint [µmol/kg]** |
| --- | --- | --- |
| 0.96 | immobile | 0.829892 |
| 0.96 | immobile | 0.793541 |
| 0.96 | immobile | 0.806132 |
| 0.96 | immobile | 1.050053 |
| 1.96 | immobile | 0.378175 |
| 1.96 | immobile | 0.375453 |
| 9.96 | immobile | 0.736853 |
| 9.96 | immobile | 0.763582 |
| 9.96 | immobile | 1.346473 |
|  |  |  |
| 0.96 | mobile | 0.636284 |
| 0.96 | mobile | 0.518136 |
| 0.96 | mobile | 0.596133 |
| 8.96 | mobile | 0.094775 |
| 8.96 | mobile | 0.049584 |
| 8.96 | mobile | 0.070491 |
| 9.96 | mobile | 0.64969 |
| 9.96 | mobile | 1.398924 |
| 9.96 | mobile | 1.395053 |
| 13.96 | mobile | 0.192397 |
| 13.96 | mobile | 0.145033 |
| 13.96 | mobile | 0.135997 |
| 13.96 | mobile | 0.22207 |
| 13.96 | mobile | 0.147068 |
| 13.96 | mobile | 0.181071 |
| 13.96 | mobile | 0.285366 |
| 13.96 | mobile | 0.129782 |
| 13.96 | mobile | 0.206161 |
| 13.96 | mobile | 0.2417 |
| 13.96 | mobile | 0.177816 |
| 13.96 | mobile | 0.18986 |
| 13.96 | mobile | 0.167322 |
| 13.96 | mobile | 0.1903 |
| 13.96 | mobile | 0.241389 |
| 13.96 | mobile | 0.230582 |
| 13.96 | mobile | 0.251747 |
| 13.96 | mobile | 0.221503 |
| 13.96 | mobile | 0.110231 |
| 13.96 | mobile | 0.236701 |
| 13.96 | mobile | 0.183987 |

**Table S8. Data on internal concentrations in 14-day experiment: Tr C**

| **Time [d]** | **mobile/immobile** | **Cint [µmol/kg]** |
| --- | --- | --- |
| 1.96 | immobile | 0.304673 |
| 8.96 | immobile | 0.289278 |
| 9.96 | immobile | 0.489761 |
|  |  |  |
| 13.96 | mobile | 0.372936 |
| 13.96 | mobile | 0.374252 |
| 13.96 | mobile | 0.405937 |
| 13.96 | mobile | 0.499365 |
| 13.96 | mobile | 0.310015 |
| 13.96 | mobile | 0.415273 |
| 13.96 | mobile | 0.464744 |
| 13.96 | mobile | 0.341824 |
| 13.96 | mobile | 0.299183 |
| 13.96 | mobile | 0.379877 |

# Raw data on feeding activity

**Table S9. Cumulative food consumption (leaf discs/*G. pulex* individual) in 14-day experiment.** Value is corrected with number of mobile individuals in the beaker.

| Beaker | 1 d | 2 d | 3 d | 4 d | 5 d | 7 d | 8 d | 9 d | 11 d | 12 d | 13 d |
| --- | --- | --- | --- | --- | --- | --- | --- | --- | --- | --- | --- |
| Control A | 0.00 | 0.20 | 0.30 | 0.40 | 0.40 | 0.40 | 0.50 | 0.50 | 0.50 | 0.50 | 0.50 |
| Solvent Control A | 0.00 | 0.30 | 0.40 | 0.50 | 0.60 | 0.70 | 0.70 | 0.90 | 0.90 | 1.00 | 1.00 |
| Control B | 0.00 | 0.00 | 0.00 | 0.00 | 0.25 | 0.50 | 0.71 | 0.71 | 0.71 | 0.71 | 0.86 |
| Solvent Control B | 0.00 | 0.00 | 0.11 | 0.33 | 0.44 | 0.44 | 0.50 | 0.63 | 0.86 | 1.14 | 1.29 |
| Control C | 0.20 | 0.30 | 0.40 | 0.40 | 0.50 | 0.80 | 0.80 | 0.90 | 1.11 | 1.33 | 1.33 |
| Solvent Control C | 0.00 | 0.10 | 0.10 | 0.10 | 0.20 | 0.50 | 0.50 | 0.50 | 0.57 | 0.71 | 0.71 |
| A1 | 0.00 | 0.00 | 0.00 | 0.00 | 0.00 | 0.00 | 0.00 | 0.00 | 0.00 | 0.00 | 0.00 |
| A2 | 0.00 | 0.00 | 0.00 | 0.00 | 0.00 | 0.00 | 0.00 | 0.14 | 0.14 | 0.29 | 0.29 |
| A3 | 0.00 | 0.00 | 0.00 | 0.00 | 0.13 | 0.14 | 0.29 | 0.57 | 0.57 | 0.71 | 0.71 |
| A4 | 0.00 | 0.00 | 0.11 | 0.22 | 0.33 | 0.38 | 0.38 | 0.50 | 0.50 | 0.50 | 0.63 |
| A5 | 0.00 | 0.00 | 0.14 | 0.14 | 0.29 | 0.29 | 0.33 | 0.33 | 0.33 | 0.40 | 0.40 |
| A6 | 0.00 | 0.00 | 0.00 | 0.00 | 0.00 | 0.00 | 0.00 | 0.00 | 0.00 | 0.00 | 0.00 |
| A7 | 0.00 | 0.00 | 0.00 | 0.00 | 0.00 | 0.00 | 0.00 | 0.00 | 0.00 | 0.14 | 0.14 |
| B1 | 0.00 | 0.00 | 0.00 | 0.00 | 0.00 | 0.00 | 0.29 | 0.43 | 0.43 | 0.43 | 0.50 |
| B2 | 0.00 | 0.00 | 0.00 | 0.00 | 0.25 | 0.25 | 0.43 | 0.57 | 0.67 | 0.83 | 1.00 |
| B3 | 0.00 | 0.00 | 0.00 | 0.00 | 0.13 | 0.13 | 0.25 | 0.25 | 0.29 | 0.33 | 0.33 |
| B4 | 0.00 | 0.00 | 0.00 | 0.00 | 0.00 | 0.00 | 0.13 | 0.25 | 0.29 | 0.29 | 0.29 |
| B5 | 0.00 | 0.00 | 0.00 | 0.00 | 0.00 | 0.00 | 0.29 | 0.43 | 0.50 | 0.50 | 0.50 |
| B6 | 0.00 | 0.00 | 0.00 | 0.00 | 0.00 | 0.00 | 0.00 | 0.00 | 0.00 | 0.13 | 0.13 |
| B7 | 0.00 | 0.00 | 0.00 | 0.00 | 0.00 | 0.14 | 0.29 | 0.33 | 0.33 | 0.60 | 0.60 |
| C1 | 0.10 | 0.10 | 0.10 | 0.10 | 0.10 | 0.10 | 0.10 | 0.11 | 0.11 | 0.13 | 0.14 |
| C2 | 0.00 | 0.00 | 0.00 | 0.00 | 0.00 | 0.00 | 0.00 | 0.00 | 0.00 | 0.00 | 0.00 |
| C3 | 0.00 | 0.00 | 0.00 | 0.00 | 0.00 | 0.00 | 0.00 | 0.00 | 0.00 | 0.00 | 0.00 |
| C4 | 0.00 | 0.00 | 0.00 | 0.00 | 0.00 | 0.00 | 0.11 | 0.22 | 0.25 | 0.25 | 0.29 |
| C5 | 0.00 | 0.00 | 0.00 | 0.00 | 0.00 | 0.00 | 0.00 | 0.00 | 0.00 | 0.00 | 0.00 |
| C6 | 0.00 | 0.00 | 0.00 | 0.00 | 0.00 | 0.00 | 0.00 | 0.00 | 0.00 | 0.00 | 0.00 |
| C7 | 0.00 | 0.00 | 0.00 | 0.00 | 0.00 | 0.00 | 0.00 | 0.00 | 0.00 | 0.00 | 0.00 |

**Table S10. Cumulative food consumption (mg/*G. pulex* individual) in 21-day experiment.** Nm denotes “not measured” in that time point. This is taken into account in the value in the next time point.

| **Controls** |  |  |  |  |  |  |  |
| --- | --- | --- | --- | --- | --- | --- | --- |
| Time [d] | A | Solv A | B | Solv B | C | Solv C |  |
| 1 | 6.16 | 10.30 | 9.13 | 6.27 | nm | nm |  |
| 2 | 12.46 | 16.34 | 17.36 | 10.62 | 9.35 | 7.40 |  |
| 5 | 20.91 | 31.09 | 29.93 | nm | nm | nm |  |
| 6 | 25.79 | 38.60 | 40.56 | 20.06 | 17.60 | 13.83 |  |
| 8 | 37.68 | 49.96 | 52.99 | 35.03 | nm | nm |  |
| 9 | 49.47 | 62.93 | 62.43 | 44.66 | 38.46 | 28.18 |  |
| 12 | nm | nm | 75.56 | 60.11 | nm | nm |  |
| 13 | 63.61 | 73.46 | 82.65 | 69.26 | 55.77 | 40.30 |  |
| 16 | 75.07 | 86.37 | nm | nm | 74.61 | nm |  |
| 19 | 91.33 | 98.10 | 92.51 | 81.77 | 86.02 | 49.80 |  |
| 21 | 106.27 | 116.46 | 113.21 | 90.31 | 101.57 | 60.41 |  |
|  |  |  |  |  |  |  |  |
| **Treatment A** | |  |  |  |  |  |  |
| Time [d] | A1 | A2 | A3 | A4 | A5 | A6 | A7 |
| 1 | 3.30 | 2.78 | 1.84 | 3.71 | 3.42 | 4.51 | 3.30 |
| 5 | 14.52 | 11.86 | 10.12 | 11.94 | 10.94 | 14.65 | 11.53 |
| 6 | 19.56 | 15.92 | 14.19 | 16.29 | 14.55 | 21.03 | 20.87 |
| 13 | 43.39 | 44.31 | 53.39 | 44.26 | 45.84 | 48.44 | 54.54 |
| 16 | 66.69 | 76.26 | nm | 83.36 | nm | nm | nm |
| 19 | 83.97 | 92.18 | 100.89 | 99.77 | 79.67 | 79.71 | 86.80 |
| 21 | 102.08 | 114.22 | 123.93 | 116.20 | 123.71 | 90.33 | 103.11 |
|  |  |  |  |  |  |  |  |
| **Treatment B** | |  |  |  |  |  |  |
| Time [d] | B1 | B2 | B3 | B4 | B5 | B6 | B7 |
| 1 | 4.01 | 2.42 | 1.63 | 1.53 | 1.71 | 1.91 | 3.06 |
| 6 | 13.33 | 12.64 | 5.21 | 11.12 | 9.45 | 8.78 | 14.93 |
| 8 | 37.77 | nm | nm | nm | nm | nm | nm |
| 9 | nm | 29.50 | nm | 37.47 | 23.49 | nm | 31.74 |
| 12 | 55.78 | 45.39 | 27.31 | 54.90 | 41.45 | 24.40 | 48.70 |
| 13 | 57.86 | 48.59 | 27.68 | 59.38 | 42.70 | 35.22 | 52.90 |
| 19 | 84.61 | 89.21 | 49.76 | 90.46 | 67.71 | 63.13 | 84.47 |
| 21 | 104.40 | 137.28 | 62.12 | 133.73 | 81.61 | 76.41 | 117.54 |
|  |  |  |  |  |  |  |  |
| **Treatment C** | |  |  |  |  |  |  |
| Time [d] | C1 | C2 | C3 | C4 | C5 | C6 | C7 |
| 2 | 4.35 | 3.63 | 3.16 | 4.03 | 3.61 | 4.60 | 4.36 |
| 6 | 13.27 | 12.04 | 7.27 | 11.26 | 8.63 | 7.89 | 10.86 |
| 9 | 21.40 | nm | nm | nm | nm | nm | nm |
| 13 | 30.66 | 24.36 | 22.34 | 19.81 | 19.09 | 15.75 | 22.40 |
| 19 | 63.01 | 32.31 | 30.94 | 31.28 | 28.64 | 28.99 | 33.53 |
| 21 | 76.45 | 42.02 | 43.95 | 44.75 | 49.04 | 36.57 | 49.17 |

# Lipid content of *G. pulex* in the end of experiment 2

**Table S11. Lipid content of immobile *Gammarus pulex* individuals in the 21-day experiment and mobile individuals in the end of experiment.**

| **Treatment A** |  |  |  |
| --- | --- | --- | --- |
| Beaker | Sampling time [d] | Mobile / Immobile | Lipid  content (%) |
| A7 | 1 | immobile | 1.635 |
| A7 | 1 | immobile | 0.821 |
| A7 | 1 | immobile | 0.638 |
| A6 | 1 | immobile | 2.631 |
| A6 | 1 | immobile | 2.471 |
| A6 | 1 | immobile | 3.436 |
| A6 | 1 | immobile | 1.066 |
| A5 | 1 | immobile | 2.041 |
| A5 | 1 | immobile | 2.883 |
| A5 | 1 | immobile | 1.263 |
| A4 | 1 | immobile | 2.036 |
| A4 | 1 | immobile | 2.567 |
| A3 | 1 | immobile | 3.683 |
| A3 | 1 | immobile | 4.412 |
| A3 | 1 | immobile | 1.679 |
| A2 | 1 | immobile | 1.092 |
| A2 | 1 | immobile | 3.020 |
| A2 | 1 | immobile | 0.892 |
| A1 | 1 | immobile | 1.023 |
| A1 | 1 | immobile | 3.147 |
| A1 | 1 | immobile | 1.741 |
| A7 | 2 | immobile | 3.228 |
| A7 | 2 | immobile | 0.920 |
| A6 | 6 | immobile | 3.054 |
| A5 | 6 | immobile | 0.616 |
| A4 | 6 | immobile | 2.861 |
| A3 | 6 | immobile | 1.480 |
| A3 | 6 | immobile | 1.009 |
| A2 | 6 | immobile | 2.456 |
| A1 | 6 | immobile | 0.701 |
| A1 | 6 | immobile | 2.723 |
| A5 | 7 | immobile | 2.588 |
| A2 | 7 | immobile | 0.924 |
| A7 | 21 | mobile | 1.404 |
| A7 | 21 | mobile | 1.097 |
| A7 | 21 | mobile | 1.360 |
| A6 | 21 | mobile | 3.225 |
| A6 | 21 | mobile | 0.752 |
| A6 | 21 | mobile | 1.417 |
| A5 | 21 | mobile | 2.588 |
| A5 | 21 | mobile | 1.462 |
| A5 | 21 | mobile | 1.017 |
| A4 | 21 | mobile | 0.768 |
| A4 | 21 | mobile | 0.808 |
| A4 | 21 | mobile | 1.315 |
| A3 | 21 | mobile | 1.001 |
| A3 | 21 | mobile | 0.606 |
| A3 | 21 | mobile | 0.733 |
| A2 | 21 | mobile | 1.087 |
| A2 | 21 | mobile | 1.417 |
| A2 | 21 | mobile | 1.740 |
| A1 | 21 | mobile | 1.561 |
| A1 | 21 | mobile | 2.172 |
| A1 | 21 | mobile | 1.933 |

| **Treatment B** |  |  |  |
| --- | --- | --- | --- |
| Beaker | Sampling time [d] | Mobile / Immobile | Lipid  content (%) |
| B7 | 1 | immobile | 2.142 |
| B7 | 1 | immobile | 0.967 |
| B6 | 1 | immobile | 0.771 |
| B6 | 1 | immobile | 3.008 |
| B5 | 1 | immobile | 0.826 |
| B5 | 1 | immobile | 1.321 |
| B4 | 1 | immobile | 1.750 |
| B4 | 1 | immobile | 1.074 |
| B4 | 1 | immobile | 2.738 |
| B3 | 1 | immobile | 2.436 |
| B3 | 1 | immobile | 3.048 |
| B3 | 1 | immobile | 2.417 |
| B2 | 1 | immobile | 2.410 |
| B2 | 1 | immobile | 3.602 |
| B2 | 1 | immobile | 4.965 |
| B1 | 1 | immobile | 2.833 |
| B1 | 1 | immobile | 3.098 |
| B1 | 2 | immobile | 2.515 |
| B7 | 2 | immobile | 0.875 |
| B6 | 2 | immobile | 0.957 |
| B5 | 2 | immobile | 3.820 |
| B4 | 2 | immobile | 2.194 |
| B1 | 2 | immobile | 1.248 |
| B7 | 13 | immobile | 0.671 |
| B5 | 13 | immobile | 2.577 |
| B4 | 13 | immobile | 2.829 |
| B2 | 13 | immobile | 2.134 |
| B1 | 13 | immobile | 0.693 |
| B7 | 21 | mobile | 1.493 |
| B7 | 21 | mobile | 0.790 |
| B7 | 21 | mobile | 2.020 |
| B6 | 21 | mobile | 0.915 |
| B6 | 21 | mobile | 0.844 |
| B6 | 21 | mobile | 1.784 |
| B5 | 21 | mobile | 0.979 |
| B5 | 21 | mobile | 2.009 |
| B5 | 21 | mobile | 0.938 |
| B4 | 21 | mobile | 1.442 |
| B4 | 21 | mobile | 1.479 |
| B4 | 21 | mobile | 3.251 |
| B3 | 21 | mobile | 2.127 |
| B3 | 21 | mobile | 1.359 |
| B3 | 21 | mobile | 1.656 |
| B2 | 21 | mobile | 1.064 |
| B2 | 21 | mobile | 1.459 |
| B2 | 21 | mobile | 1.540 |
| B1 | 21 | mobile | 1.454 |
| B1 | 21 | mobile | 0.874 |
| B1 | 21 | mobile | 1.017 |

| **Treatment C** |  |  |  |
| --- | --- | --- | --- |
| Beaker | Sampling time [d] | Mobile / Immobile | Lipid  content (%) |
| C2 | 5 | immobile | 2.456 |
| C7 | 21 | mobile | 1.305 |
| C7 | 21 | mobile | 0.582 |
| C7 | 21 | mobile | 1.176 |
| C6 | 21 | mobile | 0.675 |
| C6 | 21 | mobile | 0.660 |
| C6 | 21 | mobile | 0.724 |
| C5 | 21 | mobile | 0.559 |
| C5 | 21 | mobile | 0.703 |
| C5 | 21 | mobile | 0.769 |
| C4 | 21 | mobile | 2.677 |
| C4 | 21 | mobile | 0.619 |
| C4 | 21 | mobile | 0.606 |
| C3 | 21 | mobile | 0.555 |
| C3 | 21 | mobile | 1.742 |
| C3 | 21 | mobile | 0.702 |
| C2 | 21 | mobile | 2.331 |
| C2 | 21 | mobile | 1.113 |
| C2 | 21 | mobile | 0.621 |
| C1 | 21 | mobile | 0.653 |
| C1 | 21 | mobile | 2.254 |
| C1 | 21 | mobile | 1.824 |

| **Controls** |  |  |  |
| --- | --- | --- | --- |
| Beaker | Sampling time [d] | Mobile / Immobile | Lipid  content (%) |
| Solv A | 21 | mobile | 1.127 |
| Solv A | 21 | mobile | 0.770 |
| Solv A | 21 | mobile | 1.725 |
| Solv A | 21 | mobile | 1.298 |
| Solv A | 21 | mobile | 1.866 |
| A | 21 | mobile | 0.829 |
| A | 21 | mobile | 0.921 |
| A | 21 | mobile | 1.149 |
| A | 21 | mobile | 2.307 |
| A | 21 | mobile | 1.144 |
| Solv B | 21 | mobile | 0.973 |
| Solv B | 21 | mobile | 2.057 |
| Solv B | 21 | mobile | 0.697 |
| Solv B | 21 | mobile | 1.505 |
| Solv B | 21 | mobile | 1.032 |
| B | 21 | mobile | 1.212 |
| B | 21 | mobile | 1.910 |
| B | 21 | mobile | 2.173 |
| B | 21 | mobile | 2.715 |
| B | 21 | mobile | 1.138 |
| Solv C | 21 | mobile | 2.587 |
| Solv C | 21 | mobile | 2.027 |
| Solv C | 21 | mobile | 1.805 |
| Solv C | 21 | mobile | 0.650 |
| Solv C | 21 | mobile | 1.905 |
| C | 21 | mobile | 1.595 |
| C | 21 | mobile | 0.952 |
| C | 21 | mobile | 1.738 |
| C | 21 | mobile | 0.901 |
| C | 21 | mobile | 1.990 |

# Data on mobile fractions

**Table S12. Number of mobile and immobile individuals in the 14-day experiment.** Cint A and Cint B denotes beakers which were used to sample mobile individuals and were not used for survival modeling.

**Table S13. Number of mobile and immobile individuals in the 21-day experiment.**

# Fitting survival models with likelihood function

We also used the maximum log-likelihood function to find the final best fit values (Eqn SI-1) [[2](#_ENREF_2)]. The parameter estimates from least squares fit to all data served also as initial values for the first step (i.e. least squares fit) of fitting the models either to pulsed or constant treatments.

(SI-1)

where, *l* is the likelihood for the vector of parameters *θ* given the observations *y* and *y* is the time series of the number of survivors (*y*_0_...*y*_n_).

The likelihood function compares the observed number of death events in an observation interval with the death events predicted by the model. The log-likelihoods of the treatments were added to obtain the total likelihood. In addition, the profile of log-likelihoods was used to obtain the confidence intervals (95%) for each of the parameters [[3](#_ENREF_3)].

**Table S14. Parameter estimates for different calibration data sets.** Parameter estimates of stochastic death (SD) and individual tolerance distribution (IT) models based on fit using log-likelihood function or ordinary least squares fit.

| Summary table SD |  |  |  |  |  |  |  |  |  |  |  |  |  |  |  |  |  |  |
| --- | --- | --- | --- | --- | --- | --- | --- | --- | --- | --- | --- | --- | --- | --- | --- | --- | --- | --- |
|  |  |  |  |  |  |  |  |  |  |  |  |  |  |  |  |  |  |  |
| Likelihood |  |  |  |  |  |  |  |  |  |  |  |  |  |  |  |  |  |  |
|  | kr | ( | low CI | - | high CI | ) | kk | ( | low CI | - | high CI | ) | z | ( | low CI | - | high CI | ) |
| All data | 12881 | ( | 61.6 | - | n.d. | ) | 0.20 | ( | 0.17 | - | 0.23 | ) | 0.22 | ( | 0.18 | - | 0.25 | ) |
| Pulsed treatments | 20437 | ( | 62.1 | - | n.d. | ) | 0.19 | ( | 0.15 | - | 0.23 | ) | 0.20 | ( | 0.16 | - | 0.24 | ) |
| Constant treatments | 0.40 | ( | 0.37 | - | 0.44 | ) | 11.21 | ( | 8.75 | - | 14.1 | ) | 0.408 | ( | 0.405 | - | 0.410 | ) |
| (n.d. upper limit not found, > 20 000) | | | | | | | |  |  |  |  |  |  |  |  |  |  |  |
| Least squares |  |  |  |  |  |  |  |  |  |  |  |  |  |  |  |  |  |  |
|  | kr | ( | low CI | - | high CI | ) | kk | ( | low CI | - | high CI | ) | z | ( | low CI | - | high CI | ) |
| All data | 455 | ( | 416 | - | 494 | ) | 0.25 | ( | 0.24 | - | 0.27 | ) | 0.32 | ( | 0.32 | - | 0.32 | ) |
| Pulsed treatments | 153 | ( | 141 | - | 165 | ) | 0.24 | ( | 0.23 | - | 0.26 | ) | 0.30 | ( | 0.28 | - | 0.32 | ) |
| Constant treatments | 265 | ( | 255 | - | 274 | ) | 0.25 | ( | 0.10 | - | 0.40 | ) | 0.32 | ( | 0.30 | - | 0.35 | ) |
|  |  |  |  |  |  |  |  |  |  |  |  |  |  |  |  |  |  |  |
|  |  |  |  |  |  |  |  |  |  |  |  |  |  |  |  |  |  |  |
| Summary table IT |  |  |  |  |  |  |  |  |  |  |  |  |  |  |  |  |  |  |
|  |  |  |  |  |  |  |  |  |  |  |  |  |  |  |  |  |  |  |
| Likelihood |  |  |  |  |  |  |  |  |  |  |  |  |  |  |  |  |  |  |
|  | kr | ( | low CI | - | high CI | ) | alpha | ( | low CI | - | high CI | ) | beta | ( | low CI | - | high CI | ) |
| All data | 9.28 | ( | 5.93 | - | 18.6 | ) | 1.33 | ( | 1.19 | - | 1.49 | ) | 2.34 | ( | 1.98 | - | 2.75 | ) |
| Pulsed treatments | 9.03 | ( | 5.87 | - | 18.8 | ) | 1.37 | ( | 1.219 | - | 1.550 | ) | 2.19 | ( | 1.77 | - | 2.65 | ) |
| Constant treatments | 0.34 | ( | 0.32 | - | 0.36 | ) | 0.429 | ( | 0.427 | - | 0.432 | ) | 66 | ( | 51.3 | - | 77.6 | ) |
|  |  |  |  |  |  |  |  |  |  |  |  |  |  |  |  |  |  |  |
|  |  |  |  |  |  |  |  |  |  |  |  |  |  |  |  |  |  |  |
| Least squares |  |  |  |  |  |  |  |  |  |  |  |  |  |  |  |  |  |  |
|  | kr | ( | low CI | - | high CI | ) | alpha | ( | low CI | - | high CI | ) | beta | ( | low CI | - | high CI | ) |
| All data | 6.92 | ( | 4.19 | - | 8.79 | ) | 1.17 | ( | 1.12 | - | 1.21 | ) | 2.22 | ( | 2.04 | - | 2.40 | ) |
| Pulsed treatments | 10.03 | ( | 0.40 | - | 20 | ) | 1.19 | ( | 1.13 | - | 1.25 | ) | 2.27 | ( | 2.12 | - | 2.42 | ) |
| Constant treatments | 111 | ( | 62 | - | 161 | ) | 0.46 | ( | 0.45 | - | 0.47 | ) | 32 | ( | 18 | - | 45 | ) |
|  |  |  |  |  |  |  |  |  |  |  |  |  |  |  |  |  |  |  |
|  |  |  |  |  |  |  |  |  |  |  |  |  |  |  |  |  |  |  |
| Parameter | Units |  |  |  |  |  |  |  |  |  |  |  |  |  |  |  |  |  |
| *k*_r_ | [1/d] |  |  |  |  |  |  |  |  |  |  |  |  |  |  |  |  |  |
| *k*_k_ | [kg/µmol/d] | |  |  |  |  |  |  |  |  |  |  |  |  |  |  |  |  |
| *z* | [µmol/kg] |  |  |  |  |  |  |  |  |  |  |  |  |  |  |  |  |  |
| *alpha* | [µmol/kg] |  |  |  |  |  |  |  |  |  |  |  |  |  |  |  |  |  |
| *beta* | [ - ] |  |  |  |  |  |  |  |  |  |  |  |  |  |  |  |  |  |

References

1. Naylor, C., L. Maltby, and P. Calow, *Scope for growth in Gammarus pulex, a freshwater benthic detritivore.* Hydrobiologia, 1989. **188-189**(1): p. 517-523.

2. Jager, T., et al., *General unified threshold model of survival - a toxicokinetic-toxicodynamic framework for ecotoxicology.* Environmental Science & Technology, 2011. **45**(7): p. 2529-2540.

3. Kooijman, S.A.L.M. and J.J.M. Bedaux, *Some statistical properties of estimates of no-effect concentrations.* Water Research, 1996. **30**(7): p. 1724-1728.
